# Association between ultrafiltration variability and clinical outcomes in patients undergoing hemodialysis

**DOI:** 10.1080/0886022X.2026.2620166

**Published:** 2026-02-11

**Authors:** Seok Hui Kang, So Young Park, Yu Jeong Lim, Bo Yeon Kim, Jun Young Do

**Affiliations:** a Division of Nephrology, Department of Internal Medicine, College of Medicine, Yeungnam University, Daegu, Republic of Korea; b Department of Physiology, College of Medicine, Yeungnam University, Daegu, Republic of Korea; c Health Insurance Review and Assessment Service, Wonju, Republic of Korea

**Keywords:** Hemodialysis, ultrafiltration, dementia, survival, cardiovascular disease

## Abstract

Our study seeks to access the impact of ultrafiltration volume (UFV) variability on clinical outcomes in patients undergoing maintenance hemodialysis (HD) using a population-based cohort. This study employed a retrospective design using data derived from the national HD quality assessment initiative in Republic of Korea, which was combined with health insurance claims records (*n* = 50,583). To assess UFV variability, a linear regression model was fitted for each individual across the six measurements, and the residual standard deviation from this model was calculated. Based on this metric, patients were stratified into four quartiles representing increasing levels of UFV variability (Q1 to Q4). The 5-year survival rates for patients in Q1, Q2, Q3, and Q4 were 68.3%, 67.9%, 66.4%, and 65.8%, respectively. Multivariable analysis revealed that the hazard ratio (HR) for all-cause mortality was the highest in the Q4 group. Additionally, a spline curve using the multivariable model indicated that an increase in UFV variability, based on a median of 0.44 L/session, was linked to all-cause mortality. Multivariable Cox regression indicated that the Q4 group had a higher HR for cardiovascular events or atrial fibrillation compared to the Q1 and Q2 groups. Additionally, the Q1 group had the lowest HR for dementia among the four groups. Our study demonstrated an association between high UFV variability and various clinical outcomes, particularly all-cause mortality and dementia. These findings suggest that UFV variability could serve as a useful supplementary indicator for predicting prognosis, in addition to UFV or ultrafiltration rate.

## Introduction

Chronic kidney disease has emerged as one of the fastest growing chronic diseases in recent years. It arises from various underlying issues and can progress to end-stage kidney disease (ESKD), necessitating renal replacement therapy. Of the three types of renal replacement therapy – hemodialysis (HD), peritoneal dialysis, and kidney transplantation – HD is the most widely used globally, with 78% of patients in South Korea opting for this treatment [1]. The primary objectives of dialysis are to eliminate uremic toxins and stabilize volume status. As a short-duration, intermittent therapy, HD removes excess fluid over brief intervals, which can lead to complications such as tingling, intradialytic hypotension, dizziness, and fatigue, all of which correlate with poor patient outcomes. Consequently, ensuring a stable ultrafiltration volume (UFV) and identifying prognostic risk factors related to UFV are critical concerns for HD patients.

Traditionally, high UFV is recognized for its impact on the cardiovascular system, raising the likelihood of various cardiovascular diseases and mortality. Nevertheless, since high UFV may also indicate good nutritional status, it is difficult to definitely associate high UFV with increased cardiovascular disease risk or mortality. Thus, there is a need for additional indicators that more accurately reflect a patient’s condition and shed light on stable UFV. Variability of UFV could serve as such an indicator. Cases of high UFV that are stable may differ from those of low UFV that unstable in terms of UFV variability, leading to potentially differing prognostic outcomes. Zhang et al. investigated the variability in UFV and all-cause mortality across 283 patients undergoing HD [2]. However, the clinical significance of UFV variability is generally underexplored. Therefore, this study seeks to access the impact of UFV variability on clinical outcomes in patients undergoing maintenance HD using a population-based cohort.

## Methods

### Data source and study population

This study employed a retrospective design using data derived from the national HD quality assessment initiative in Republic of Korea, which was combined with health insurance claims records [3,4]. The quality assessment program has been implemented in multiple cycles since 2010, and for this study, data were extracted from the sixth (March–August 2018) and seventh (October–March 2021) cycles.

**Figure 1.**
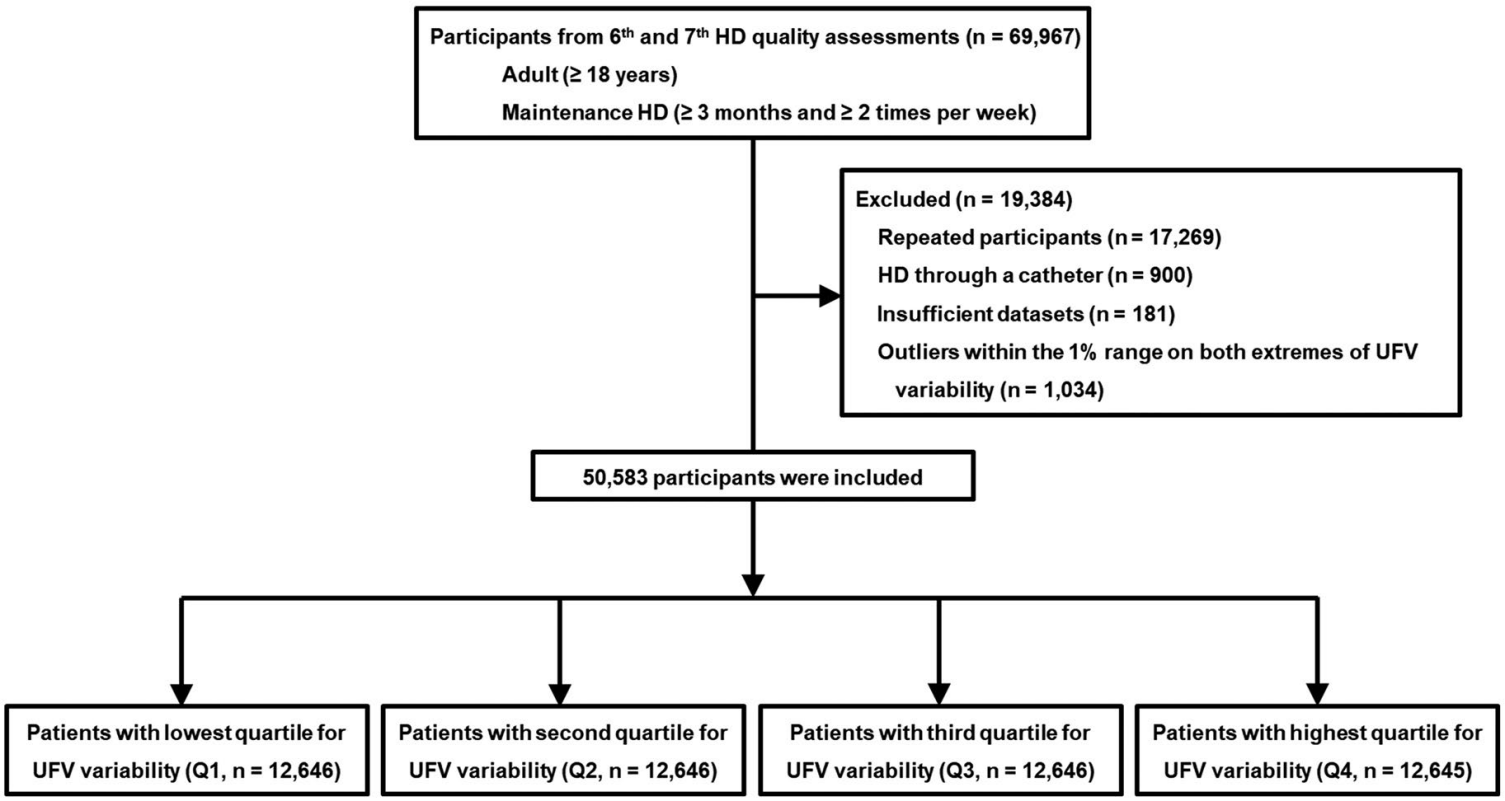
Study flowchart. *Abbreviations:* HD, hemodialysis; UFV, ultrafiltration volume.

Eligible patients were adult (≥ 18 years old) who had been receiving regular HD for more than 3 months, at a frequency of at least twice per week. The dataset included both clinical information obtained through quality assessments and administrative claims. Among the initial pool of 69,967 patients, we excluded duplicate participants (*n* = 17,269), individuals using a catheter for dialysis access (*n* = 900), those with incomplete clinical data (*n* = 181), and those with outlier values in UFV variability falling within the top or bottom 1% (*n* = 1,034) (Figure 1). No patients were excluded due to missing UFV data. As a results, 50,583 patients were included in the final cohort for analysis. The study protocol was reviewed and approved by the Institutional Review Board of Yeungnam University Medical Center (approval number: YUMC 2023-12-012). Since the data were anonymized and obtained retrospectively, informed consent was not required. The study was conducted in accordance with the principles of the Declaration of Helsinki.

### Clinical and demographic variables

Patient characteristics, including age, sex, HD duration, and vascular access type, were documented at baseline. Laboratory parameters assessed monthly over a six-month period included hemoglobin (g/dL), body mass index (kg/m^2^), Kt/V_urea_ (calculated using the Daugirdas equation [5]), serum albumin (g/dL), calcium (mg/dL), phosphorus (mg/dL), and creatinine (mg/dL). Mean values from monthly tests were used for analysis. Monthly UFV values were recorded from either Monday or Tuesday sessions, and the mean of six monthly values was used to represent each patient’s UFV. To assess UFV variability, a linear regression model was fitted for each individual across the six measurements, and the residual standard deviation from this model was calculated. Based on this metric, patients were stratified into four quartiles representing increasing levels of UFV variability (Q1 to Q4). Furthermore, weight-adjusted UFV (wUFV) was calculated as UFV divided by post-dialysis body weight. Moreover, wUFV variability was defined as the residual standard deviation from a linear regression model using six wUFVs. Weight-adjusted quartiles (aQ1–aQ4) were defined based on wUFV variability.

In addition to the residual standard deviation, we calculated the coefficient of variation (CV) of UFV to account for inter-individual variability in the mean UFV value. The CV was calculated by dividing the standard deviation of six UFV measurements by the corresponding mean UFV. This adjustment enables the interpretation of UFV variability in relation to the mean UFV, thereby offering a more standardized assessment of variability among patients with different baseline UFV values.

Medication usage during the assessment period was identified *via* claims data (see Table S1 for code definitions). We assessed the use of renin–angiotensin system blockers (RASB), statins, clopidogrel, aspirin, and anti-hypertensive drugs. Use was defined as at least one prescription issued during the evaluation window. To evaluate comorbid status, we assessed claims data from the 12 months preceding the HD assessment. The Charlson Comorbidity Index (CCI) was used to quantify overall disease burden [6,7]. Specific conditions including myocardial infarction (MI), congestive heart failure (CHF), and atrial fibrillation (Afib) were identified using ICD-10 codes.

### Clinical outcomes and follow-up

Patients were followed from the end of the HD quality assessment until June 2024. Outcomes of interest included all-cause mortality, incident cardiovascular events (CVEs), newly diagnosed Afib, and dementia. Mortality data were obtained from national death registry linkage. CVEs were defined as a composite of MI, stroke, or revascularization procedures [8], regardless of subsequent survival. Incident cases of Afib and dementia were identified using ICD-10 codes: I48 for Afib, and F00-03, G30, and G31 for dementia. To ensure incident case identification, patients with relevant diagnoses during the six-month HD assessment or in the preceding year were excluded from analyses of Afib and dementia incidence. Patients who transitioned to peritoneal dialysis or underwent kidney transplantation were censored at the time of modality change unless an event had already occurred.

### Statistical analysis

All analyses were performed using SAS Enterprise Guide version 7.1 and R version 3.5.1. Continuous variables are described as mean ± standard deviation and compared using one-way analysis of variance with Tukey’s *post hoc* correction. Categorical variables are presented as frequencies and proportions, and comparisons were made using the chi-square test or Fisher’s exact test as appropriate. Survival probabilities were estimated using Kaplan–Meier curves, and differences across quartiles were tested using the log-rank method. Cox proportional hazard models were constructed to estimate adjusted hazard ratios (HRs) and 95% confidence intervals (CIs) for each outcome. Multivariable models included the following covariates: age, vascular access type, sex, body mass index, diabetes, HD duration, CCI score, mean UFV, Kt/V_urea_, hemoglobin, serum albumin, calcium, phosphorus, creatinine, and use of RASB, statins, clopidogrel, aspirin, and anti-hypertensive drugs. Comorbid MI, CHF, and Afib were also adjusted. All Cox models used the enter selection method.

Subgroup analyses were conducted based on median splits of age, HD durations, and CCI score, as well as sex and mean UFV categories. Additional analyses were performed by stratifying patients with right-sided (ICD-10: I50.0) and left-sided heart failure (ICD-10: I50.1), examining differential associations with clinical outcomes. To explore non-linear associations between UFV variability and outcomes, we applied restricted cubic spline models adjusted for the same covariates included in the multivariable Cox models.** **We have evaluated the C-index. The Cox proportional hazards model’s discriminative ability was evaluated using Harrell’s concordance index, where values closer to 1.0 signify better discrimination. Furthermore, baseline characteristics, Kaplan-Meier curves, and findings of Cox regression analyses were compared using adjusted quartiles based on wUFV variability.

To balance the baseline characteristics between the Q1 and Q4 groups, we calculated propensity scores utilizing logistic regression models and the following variables: age, sex, body mass index, type of vascular access, CCI score, HD vintage, diabetes, UFV, Kt/V_urea_, levels of hemoglobin, albumin, creatinine, phosphorus, and calcium, and the administration of aspirin, clopidogrel, RASB, antihypertensive drugs, or statins, the presence of MI, CHF, or Afib. Participants in the Q4 group were matched with participants in the Q1 group using 1:1 nearest neighbor matching without replacement and with a matching tolerance (caliper) of 0.1, based on propensity scores. Statistical significance was defined as a *p* < 0.05.

## Results

### Baseline characteristics

In the first, second, third, and fourth quartiles, the patient count were 12,646, 12,646, 12,646, and 12,645, respectively (Table 1). The residual SD of UFV variability in Q1, Q2, Q3, and Q4 was 0.22 ± 0.06, 0.38 ± 0.04, 0.52 ± 0.05, and 0.77 ± 0.14 L/session, respectively. The patients in Q1 were older than those in the other groups. Additionally, this group had a higher Kt/V_urea_ and a greater proportion of statin usage. However, this group exhibited lower UFV and serum creatinine levels, as well as a lower proportion of males, diabetes, and the use of RASB or anti-hypertensive drugs compared to the other groups. The Q3 and Q4 groups had lower hemoglobin levels and higher aspirin usage than the other groups.

**Table 1.** Baseline characteristics.

	Q1 (*n* = 12,646)	Q2 (*n* = 12,646)	Q3 (*n* = 12,646)	Q4 (*n* = 12,645)	*p*-value
Age (years)	62.5 ± 12.9	62.4 ± 12.7	62.1 ± 12.8	60.7 ± 13.0^abc^	<0.001
Sex (male, %)	6,895 (54.5)	7,226 (57.1)	7,791 (61.6)	8,665 (68.5)	<0.001
Hemodialysis vintage (months)	64 ± 68	67 ± 69^a^	68 ± 69^a^	66 ± 66	<0.001
Body mass index (kg/m^2^)	22.8 ± 2.7	22.7 ± 3.6	22.7 ± 3.6	22.9 ± 3.6^bc^	<0.001
Diabetes (%)	5,253 (41.5)	5,423 (42.9)	5,569 (44.0)	5,898 (46.6)	<0.001
CCI score	8.8 ± 2.9	8.8 ± 2.9	8.9 ± 2.9	8.9 ± 2.8^a^	0.006
Arteriovenous fistula (%)	10,894 (86.1)	10,884 (86.1)	10,904 (86.2)	10,951 (86.6)	0.613
Kt/V_urea_	1.59 ± 0.28	1.59 ± 0.28	1.57 ± 0.27^ab^	1.53 ± 0.26^abc^	<0.001
Ultrafiltration volume (L/session)	2.27 ± 1.01	2.30 ± 0.86^a^	2.31 ± 0.80^a^	2.35 ± 0.77^abc^	<0.001
Hemoglobin (g/dL)	10.7 ± 0.7	10.7 ± 0.7	10.6 ± 0.7^a^	10.6 ± 0.7^ab^	<0.001
Serum albumin (g/dL)	4.01 ± 0.32	4.01 ± 0.33	4.01 ± 0.32	4.01 ± 0.34	0.633
Serum phosphorus (mg/dL)	4.99 ± 1.20	4.99 ± 1.19	4.96 ± 1.18	5.01 ± 1.26^c^	0.029
Serum calcium (mg/dL)	8.9 ± 0.7	8.9 ± 0.7	8.9 ± 0.7	8.9 ± 0.7	0.271
Serum creatinine (mg/dL)	9.4 ± 2.7	9.5 ± 2.6^a^	9.6 ± 2.6^a^	9.8 ± 2.7^abc^	<0.001
Use of RASB (%)	7,961 (63.0)	8,212 (64.9)	8,443 (66.8)	8,874 (70.2)	<0.001
Use of aspirin (%)	5,867 (46.4)	6,137 (48.5)	6,345 (50.2)	6,344 (50.2)	<0.001
Use of clopidogrel (%)	3,342 (26.4)	3,262 (25.8)	3,270 (25.9)	3,259 (25.8)	0.590
Use of statins (%)	6,496 (51.4)	6,377 (50.4)	6,278 (49.6)	6,038 (47.8)	<0.001
Use of antihypertensive drug	10,458 (82.7)	10,635 (84.1)	10,781 (85.3)	11,092 (87.7)	<0.001
MI or CHF (%)	7,356 (58.2)	7,372 (58.3)	7,475 (59.1)	7,370 (58.3)	0.401
Atrial fibrillation (%)	1,567 (12.4)	1,590 (12.6)	1550 (12.3)	1,479 (11.7)	0.168
Dementia (%)	1,090 (8.6)	1,065 (8.4)	1,036 (8.2)	1,040 (8.2)	0.586
SD of ultrafiltration volume	0.31 ± 0.13	0.46 ± 0.11^a^	0.59 ± 0.12^ab^	0.85 ± 0.18^abc^	<0.001
CV of ultrafiltration volume	0.18 ± 0.17	0.24 ± 0.15^a^	0.30 ± 0.16^ab^	0.40 ± 0.18^abc^	<0.001

Data are expressed as means ± standard deviation for continuous variables and as numbers (percentages) for categorical variables. *p*-values were tested using one-way analysis of variance, followed by the Tukey’s post-hoc test. Pearson’s χ^2^ test was performed for categorical variables.

^a^*p* < 0.05 vs. Q1, ^b^*p* < 0.05 vs. Q2, ^c^*p* < 0.05 vs. Q3.

***Abbreviations:*** Q1, first quartile of variability in ultrafiltration volume; Q2, second quartile; Q3, third quartile; Q4, fourth quartile; CCI, Charlson comorbidity index; CHF, congestive heart failure; MI, myocardial infarction; RASB, renin–angiotensin system blocker; SD, standard deviation; CV, coefficient of variation.

### All-cause mortality by group

**Figure 2.**
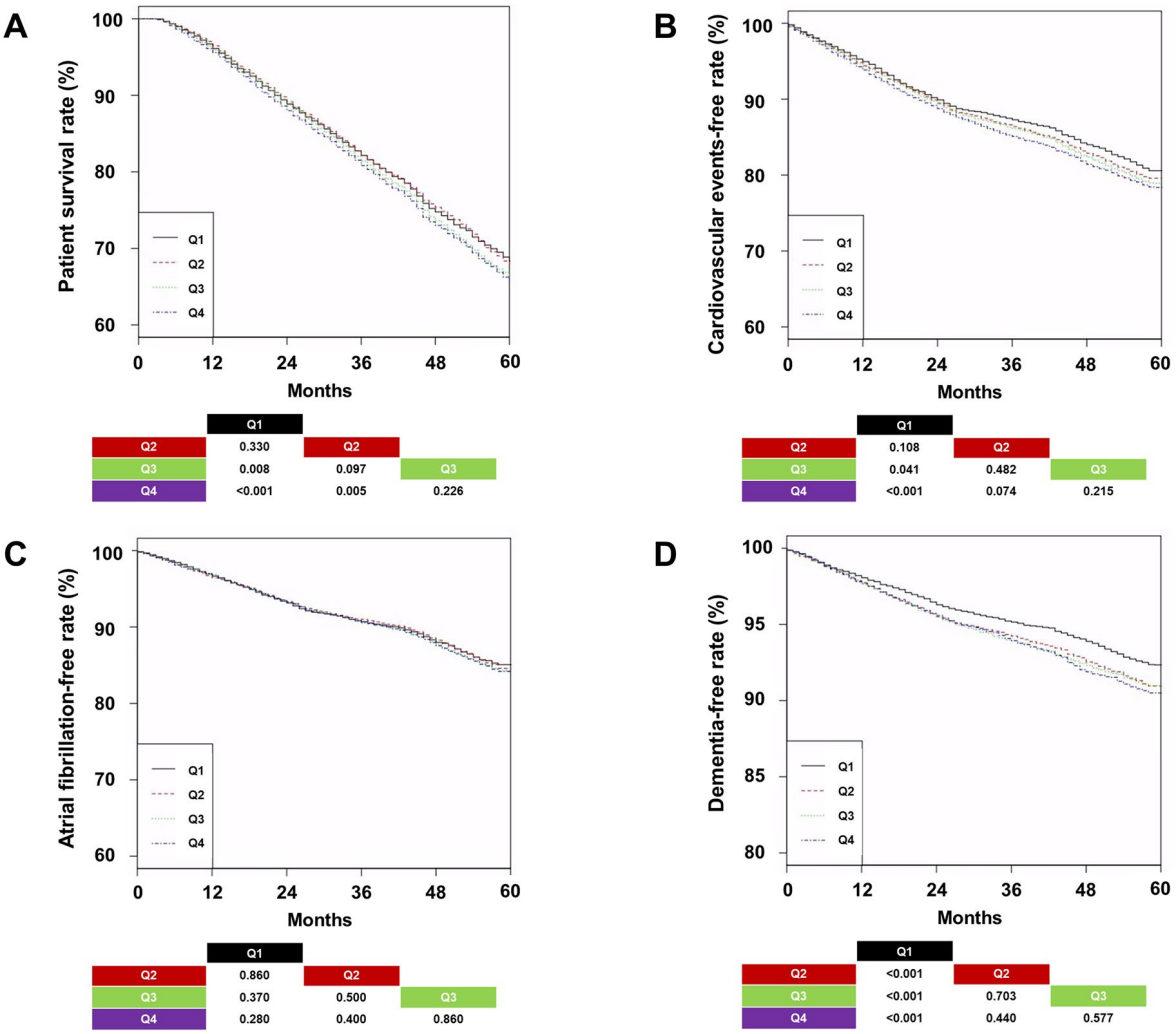
Kaplan–Meier curves of patient survival, cardiovascular events, atrial fibrillation, and dementia according to quartile. (A) Patient survival. (B) Cardiovascular events. (C) Atrial fibrillation. (D) Dementia. *p*-values for pairwise comparisons with log-rank tests were added to the bottom of the graph. *Abbreviations:* Q1, first quartile; Q2, second quartile; Q3, third quartile; Q4, fourth quartile.

The follow-up durations for the Q1, Q2, Q3, and Q4 groups were 48 ± 20, 49 ± 20, 50 ± 21, and 51 ± 21 months, respectively. At the end of follow-up, patient outcomes included survival, death, transfer to peritoneal dialysis, or kidney transplantation as follows: 8,399 (66.4%), 3,615 (28.6%), 15 (0.1%), and 617 (4.9%) for Q1; 8,188 (64.7%), 3,854 (30.5%), 23 (0.2%), and 581 (4.6%) for Q2; 7,896 (62.4%), 4,106 (32.5%), 23 (0.2%), and 621 (4.9%) for Q3; and 7,654 (60.5%), 4,309 (34.1%), 25 (0.2%), and 657 (5.2%) for Q4, respectively (*p* < 0.001). The proportion of deaths was highest in the Q4 group among the four groups. The 5-year survival rates for patients in Q1, Q2, Q3, and Q4 were 68.3%, 67.9%, 66.4%, and 65.8%, respectively (Figure 2A). Multivariable analysis revealed that the HR for all-cause mortality was the highest in the Q4 group (Table 2). Additionally, a spline curve using the multivariable model indicated that an increase in UFV variability, based on a median of 0.44 L/session, was linked to all-cause mortality (Figure S1A).

**Table 2.** Quartile of ultrafiltration volume and HR of clinical outcomes.

	Univariable		Multivariable	
	HR (95% CI)	*p*-value	HR (95% CI)	*p*-value
All-cause mortality				
Ref: Q1				
Q2	1.02 (0.98–1.07)	0.328	1.00 (0.95–1.05)	0.975
Q3	1.07 (1.02–1.12)	0.005	1.03 (0.98–1.08)	0.189
Q4	1.10 (1.05–1.15)	<0.001	1.12 (1.06–1.17)	<0.001
Ref: Q2				
Q3	1.04 (0.99–1.09)	0.062	1.03 (0.98–1.08)	0.192
Q4	1.07 (1.03–1.12)	0.001	1.12 (1.06–1.17)	<0.001
Ref: Q3				
Q4	1.03 (0.99–1.07)	0.188	1.08 (1.03–1.13)	0.001
Cardiovascular events				
Ref: Q1				
Q2	1.07 (0.99–1.16)	0.070	1.05 (0.97–1.14)	0.233
Q3	1.10 (1.02–1.19)	0.012	1.07 (0.99–1.16)	0.106
Q4	1.16 (1.07–1.25)	<0.001	1.14 (1.05–1.24)	0.002
Ref: Q2				
Q3	1.03 (0.95–1.11)	0.479	1.02 (0.94–1.10)	0.670
Q4	1.08 (1.01–1.16)	0.041	1.08 (1.01–1.17)	0.048
Ref: Q3				
Q4	1.05 (0.98–1.13)	0.181	1.06 (0.98–1.16)	0.118
Atrial fibrillation				
Ref: Q1				
Q2	1.01 (0.93–1.09)	0.852	1.00 (0.92–1.09)	0.989
Q3	1.04 (0.96–1.12)	0.386	1.04 (0.95–1.13)	0.408
Q4	1.04 (0.96–1.13)	0.298	1.10 (1.01–1.20)	0.024
Ref: Q2				
Q3	1.03 (0.95–1.11)	0.494	1.04 (0.95–1.13)	0.396
Q4	1.03 (0.96–1.12)	0.391	1.10 (1.01–1.20)	0.022
Ref: Q3				
Q4	1.01 (0.93–1.09)	0.863	1.06 (0.98–1.15)	0.143
Dementia				
Ref: Q1				
Q2	1.21 (1.09–1.34)	<0.001	1.21 (1.08–1.35)	<0.001
Q3	1.23 (1.11–1.36)	<0.001	1.24 (1.12–1.39)	<0.001
Q4	1.27 (1.15–1.41)	<0.001	1.36 (1.22–1.52)	<0.001
Ref: Q2				
Q3	1.02 (0.92–1.12)	0.713	1.03 (0.93–1.14)	0.556
Q4	1.05 (0.96–1.16)	0.291	1.13 (1.02–1.25)	0.024
Ref: Q3				
Q4	1.03 (0.94–1.14)	0.491	1.09 (0.99–1.21)	0.091

Multivariable analysis was adjusted for age, sex, body mass index, vascular access type, diabetes, hemodialysis vintage, Charlson Comorbidity Index score, ultrafiltration volume, Kt/V_urea_, levels of hemoglobin, serum albumin, serum creatinine, serum phosphorus, and serum calcium, use of renin–angiotensin system blockers, statins, clopidogrel, aspirin, or anti-hypertensive drugs, presence of myocardial infarction or congestive heart failure, and atrial fibrillation.

*Abbreviations:* Q1, first quartile of variability in ultrafiltration volume; Q2, second quartile; Q3, third quartile; Q4, fourth quartile; CI, confidence interval; HR, hazard ratio.

### Other clinical outcomes analyzed according to group

We also evaluated the association between CVE, Afib, or dementia and UFV variability. The 5-year CVE-free rates in the Q1, Q2, Q3, and Q4 were 80.6%, 79.6%, 78.9%, and 78.4%, respectively (Figure 2B). The 5-year Afib-free rates in the Q1, Q2, Q3, and Q4 were 85.1%, 84.6%, 84.3%, and 84.2%, respectively (Figure 2C). The 5-year dementia-free rates in the Q1, Q2, Q3, and Q4 were 92.3%, 91.0%, 91.0%, and 90.5%, respectively (Figure 2D). Multivariable Cox regression indicated that the Q4 group had a higher HR for CVE or Afib compared to the Q1 and Q2 groups. Additionally, the Q1 group had the lowest HR for dementia among the four groups. Spline curves demonstrated trends similar to those observed in the Cox regression (Figure S1B-D).

Some degree of overlap among covariates may exist; therefore, we calculated the variance inflation factor (VIF) in the Cox regression analyses. In the models for all-cause mortality, CVE, Afib, and dementia, the VIF values for the covariates ranged between 1.04 and 1.84 (e.g., age 1.39–1.49, body mass index 1.32–1.36, Kt/V 1.60–1.67, creatinine 1.72–1.84, RASBs 1.48–1.53, antihypertensive drugs 1.47–1.52). These results demonstrate that all variables were significantly below the conventional threshold of 10, confirming that multicollinearity was not a concern in our analyses.

The C-indices (95% CI) for all-cause mortality, CVE, Afib, or dementia were 0.75 (0.74–0.76), 0.66 (0.65–0.67), 0.65 (0.64–0.66), and 0.72 (0.70–0.73), respectively. These results reveal that the model for all-cause mortality exhibited good discriminative ability, the model for dementia demonstrated moderate discrimination, and the models for CVE and Afib demonstrated relatively lower predictive performance. The C-indices exhibited a consistent pattern corresponding to the statistical significance identified in the Cox regression analyses.

### Subgroup analyses

To determine the specific groups in which the effects of UFV variability were more pronounced, we conducted subgroup analyses based on sex, age (< 65 or ≥ 65 years), HD vintage (< 41 or ≥ 41 months, defined as the median HD vintage), CCI score (< 7 or ≥ 7, defined as the median CCI score), and mean UFV (≤ 1, 1–2, 2–3, or > 3 L/session) (Table S2).

The hazard effect for all-cause mortality in Q4 was similar across subgroups by sex, age, and HD vintage. Although significant interactions were not observed, high CCI and ≤ 3 L/session UFV subgroups had a prominent effect on all-cause mortality. Subgroup analyses for CVE or Afib did not reveal significant differences across the subgroups (Tables S3 and S4).

The Q1 group also demonstrated better dementia outcomes across both sex and age subgroups (Table S5). High CCI or 1–3 L/session UFV subgroups exhibited prominent protective effect in the Q1 group despite non-significant interactions. In the long HDV group, the HR for dementia increased with UFV variability, demonstrating significant interactions compared to the short HDV subgroup.

The number of patients with right HF was 3,667, while the number with left HF was 569. Table S6 presents these results. Subgroup analyses regarding right- or left-sided HF did not show statistically significant differences in outcomes, nor was any meaningful interaction between the side of HF and clinical outcomes observed.

### Association between wUFV variability values and clinical outcomes

The mean wUFV variability values for the Q1, Q2, Q3, and Q4 groups were 0.005 ± 0.002, 0.008 ± 0.002, 0.010 ± 0.003, and 0.014 ± 0.004, respectively (*p* < 0.001). The correlation coefficient between UFV variability and wUFV variability was 0.797 (*p* < 0.001), signifying a significant correlation between the two metrics. In Cox regression analyses, each 0.001 increase in wUFV variability was associated, in univariable analysis, with hazard ratios of 1.03 (95% CI: 1.02–1.03; *p* < 0.001) for all-cause mortality, 1.02 (1.01–1.02; *p* < 0.001) for CVE, 1.00 (0.99–1.01; *p* = 0.250) for Afib, and 1.03 (1.02–1.04; *p* < 0.001) for dementia. In multivariable analyses, each 0.001 increase in wUFV variability was significantly correlated with all-cause mortality (HR: 1.02; 95% CI: 1.01–1.02; *p* < 0.001), CVE (HR: 1.01; 95% CI: 1.01–1.02; *p* < 0.001), and dementia (HR: 1.03; 95% CI: 1.02–1.04; *p* < 0.001), but not with Afib (HR: 1.01; 95% CI: 0.99–1.01; *p* = 0.076). These findings indicate that higher wUFV variability is significantly correlated with increased risks of mortality, CVE, and dementia in patients undergoing maintenance HD.

Furthermore, we defined quartiles (aQ1-aQ4) of variability using wUFV. Table S7 shows baseline characteristics based on quartiles of variability using wUFV. Values of wUFV variability (× 10^3^) were 3.61 ± 1.01 in aQ1, 6.22 ± 0.65 in aQ2, 8.65 ± 0.80 in aQ3, and 13.20 ± 2.71 in aQ4. The patients in aQ1 were younger than those in the other groups. This group had a higher UFV and higher serum phosphorus and creatinine levels, as well as a greater proportion of males, increased prevalence of diabetes, and greater use of clopidogrel or statins. Moreover, this group had a lower Kt/V_urea_ value and lower use of RASB, aspirin, or anti-hypertensive drugs than the other groups.

We also evaluated the association between patient survival, CVEs, Afib, or dementia and quartiles of wUFV variability. The 5-year patient survival rates in aQ1, aQ2, aQ3, and aQ4 were 69.8%, 68.5%, 66.6%, and 63.7%, respectively (Figure S2A). The 5-year CVE-free rates in aQ1, aQ2, aQ3, and aQ4 were 80.2%, 79.7%, 79.2%, and 78.4%, respectively (Figure S2B). The 5-year Afib-free rates in aQ1, aQ2, aQ3, and aQ4 were 84.5%, 84.8%, 84.5%, and 84.3%, respectively (Figure S2C). Moreover, the 5-year dementia-free rates in aQ1, aQ2, aQ3, and aQ4 were 92.5%, 91.3%, 90.9%, and 90.1%, respectively (Figure S2D). Multivariable Cox regression analyses showed the same trends as those using unadjusted quartiles (Table S8).

### Association between CV values and clinical outcomes

The mean CV of UFV in the Q1, Q2, Q3, and Q4 groups was 0.18 ± 0.17, 0.24 ± 0.15, 0.30 ± 0.16, and 0.40 ± 0.18, respectively (*p* < 0.001). In Cox regression analyses, each 1-unit increase in the CV of UFV variability was significantly associated, in univariable analysis, (HR: 1.61; 95% CI, 1.49–1.74; *p* < 0.001) for all-cause mortality (HR: 1.26; 95% CI: 1.10–1.45; *p* < 0.001) for CVE (HR: 1.14; 95% CI: 0.98–1.32; *p* = 0.087) for Afib, and (HR: 2.01; 95% CI: 1.69–2.38; *p* < 0.001) for dementia. In multivariable analyses, each 1-unit increase in CV was significantly associated with all-cause mortality (HR: 1.40; 95% CI: 1.24–1.58; *p* < 0.001), CVE (HR: 1.43; 95% CI: 1.16–1.76; *p* < 0.001), Afib (HR: 1.48; 95% CI: 1.19–1.83; *p* < 0.001), and dementia (HR: 1.68; 95% CI: 1.29–2.17; *p* < 0.001), These findings indicate that higher CV of UFV variability is significantly associated with four clinical outcomes among patients undergoing maintenance HD.

### Analyses using propensity score matching cohort

Table S9 and Figure S3 present the matched cohort and the distribution of the propensity score. Differences in most variables, including age, were attenuated after propensity score matching, and the distribution of propensity scores was similar between the two groups after matching. Cox regression analyses were conducted between Q1 and Q4 groups after matching (Table S10). In multivariable analyses, Q4 exhibited significantly higher HR than Q1 across four outcomes.

## Discussion

Our study used data from 50,583 HD patients in the nationwide HD quality assessment program and calculated UFV variability using 6 months of monthly collected UFV data, which were categorized into quartiles. Our results showed that patient survival rates decreased as quartiles increased, with a similar trend observed in the Cox analysis. For CVE and Afib, the Q4 group exhibited a higher risk compared to Q1. Notably, the risk of dementia was lower in groups with less UF variability. In the subgroup analysis, higher all-cause mortality in Q4 and lower dementia risk in Q1 were more pronounced in patients with a higher CCI or a mean UFV < 3 L/session, with a longer HD vintage showing a stronger association with dementia.

In patients undergoing HD, UFV is achieved within a limited timeframe, and its increase can impact various clinical outcomes. High UFV influences hospitalization rates and mortality through cardiovascular complications such as intradialytic hypotension and myocardial stunning [9–12]. Additionally, high UFV often indicates a state of volume overload, which can adversely affect prognosis through mechanisms such as left ventricular hypertrophy and hypertension. However, relying solely on UFV to assess volume status or predict prognosis has several limitations. First, UFV is strongly correlated with inter-dialytic weight gain, which, in turn, is positively correlated with nutritional status [13]. This makes it challenging to independently assess the relationship between UFV and prognosis in cases where UFV is high but nutritional status is good, or UFV is low but nutritional status is poor. Secondly, some patients who require high UFV may not achieve adequate ultrafiltration due to intradialytic complications or improper clinical settings, resulting in low UFV despite being in a state of volume overload [14]. Conversely, some patients who require low UFV may be misinterpreted as having volume overload due to incorrect dry weight settings that lead to high UFV.

Considering the relationship between UFV and nutritional status, as well as the impact of dry weight settings on volume status within the same UFV, UFV variability – which reflects stable fluid intake, UFV, and dry weight – could serve as an additional indicator. UFV variability, even within the same UFV range, introduces another fluctuation factor that may influence patient outcomes. Zhang et al. in a study of 283 HD patients, demonstrated that UFV variability is associated with all-cause mortality [2]. Additionally, a smaller study found that UFV variability was linked to left ventricular hypertrophy, left ventricular dysfunction, higher shear stress, and endothelial injury, even within the same UFV range [15]. Our findings are consistent with these studies, showing a trend of increasing all-cause mortality with higher UFV variability quartiles. Although the association with CVE or Afib was less statistically significant, a similar trend was observed. Notably, we also demonstrated an association between UFV variability and dementia. Our study highlights relationships between UFV variability and both Afib and dementia, supported by Flythe et al. who reported a higher Afib incidence in HD patients with a high UFV rate among 15,141 subjects [16]. Furthermore, Shi et al. found that high UFV increases the risk of cerebral small-vessel disease, a known risk factor for dementia [17]. While these studies did not directly analyze UFV variability, our findings suggest that similar mechanisms may underlie the increased risks of Afib and dementia associated with UFV variability.

The association between UFV variability and incident dementia was clearly observed in our study. Although the precise biological mechanisms linking UFV variability to cognitive impairment remain unclear, emerging physiological evidence regarding UFV variability and cardiovascular responses provides plausible explanations supporting this association. UFV variability ultimately reflects large fluctuations in solute and fluid status during the interdialytic period. Such instability may impose an additional hemodynamic burden on the cardiovascular system beyond the absolute UFV itself. Sági et al. found that only patients with high UFV variability exhibited a significant increase in post-dialysis pulse wave velocity, indicating an acute increase in arterial stiffness following HD [15]. This suggests that recurrent exposure to marked UFV fluctuations can cause short-term vascular stiffening. Moreover, repeated episodes of inadequate UFV in patients with high UFV variability have been associated with intradialytic hypotension. These hemodynamic insults, including UFV variability-driven arterial stiffness, recurrent intradialytic hypotension, and insufficient fluid removal, contribute to chronic cardiovascular stress. In addition, UFV variability may coexist with other detrimental factors frequently observed in patients undergoing HD, including malnutrition, fluctuations in hemoglobin or serum albumin levels, and variability in solute clearance. A combination of these perturbations could adversely affect cerebral perfusion or contribute to microvascular injury, ultimately promoting vascular-origin cognitive decline or dementia. Therefore, the observed association between UFV variability and dementia in our study may be biologically plausible; however, further mechanistic and longitudinal studies are warranted to validate these potential pathways and establish causality.

In our study, subgroup analysis revealed a statistically significant interaction between HD vintage and dementia. For other outcomes, although statistical significance was lower, all-cause mortality showed a stronger trend in patients with a high CCI or mean UFV < 3 L/session. This aligns with previous studies comparing ultrafiltration rate and all-cause mortality. Assimon et al. reported a larger effect size for all-cause mortality in patients with long HD vintage [18], while Sági et al. found more significant results for UFV variability and all-cause mortality in subgroups of older patients, males, and those with comorbidities [15]. These populations are generally more susceptible to cardiovascular disease, making them more likely to be affected by UFV or its variability, ultimately influencing their outcomes. In our study, similar trends were observed for all-cause mortality and dementia; however, distinct trends were not apparent for CVE or Afib in the subgroup analysis.

In addition, subgroup analyses regarding right- or left-sided HF did not show statistically significant differences in outcomes. The lack of statistical significance is likely attributable to limitations in the accuracy of ICD-10 coding. In our study, the identification of HF was evaluated using a subset of specific diagnostic codes. However, in clinical practice, physicians often do not specify the side of HF when entering diagnostic codes, leading to a significant decrease in the number of classified cases. This, in turn, may have limited the power of the subgroup analysis, contributing to the non-significant findings. The apparent predominance of right HF over left HF in our data likely reflects coding techniques rather than true pathophysiologic disparities. In the national claims database, ‘right heart failure (I50.0)’ is widely utilized as a general diagnostic code for congestive or unspecified HF, or conditions such as diastolic dysfunction according to volume overloading, whereas left HF (I50.1) may be less frequently coded than right HF. In our study, echocardiographic parameters were unavailable to confirm the anatomical subtype, and the observed discrepancy likely stems from administrative coding patterns rather than actual clinical prevalence. Therefore, the prevalence and analytic results for right and left HF may lack complete reliability. Future studies should integrate echocardiographic findings that accurately delineate the anatomical region and features of cardiac dysfunction to ensure accurate classification of HF type.

Vericiguat is a novel oral soluble guanylate cyclase (sGC) stimulator that enhances the cyclic guanosine monophosphate pathway by directly stimulating sGC through a binding site independent of nitric oxide while also sensitizing sGC to endogenous nitric oxide by stabilizing its binding [19]. In a phase 3 clinical trial conducted in patients with HF with reduced ejection fraction (<45%) who were already receiving guideline-directed medical therapy, vericiguat demonstrated a statistically significant reduction in both the primary outcome (a composite of cardiovascular death or first hospitalization for HF) and the composite of all-cause mortality or hospitalization for HF [20]. Furthermore, a recent meta-analysis indicated that vericiguat outperformed standard care in reducing cardiovascular death or HF hospitalization; however, it was less effective than SGLT2 inhibitors and exhibited a neutral effect compared to sacubitril/valsartan [21]. Based on this evidence, vericiguat has been recommended as a second-line agent for patients with worsening HF, particularly those requiring intravenous diuretics or with recent hospitalizations due to HF [22]. These findings suggest that vericiguat may offer potential benefits in improving symptoms or outcomes for HF patients receiving dialysis. However, in our study, various obstacles hindered the inclusion of vericiguat as a variable. First, previous studies regarding this medication have focused exclusively on patients with eGFR ≥ 15 mL/min/1.73 m^2^, leaving a gap in research regarding its effects on patients with eGFR < 15 mL/min/1.73 m^2^ or those on dialysis [19]. Currently, there is no established evidence supporting its use in this population, and its use is not recommended, especially in South Korea [23]. Second, this study assessed medication use based on two 6-month prescription intervals: March to August 2018 and October 2020 to March 2021, with outcomes monitored until June 2024. However, as this medication has only been reimbursed in South Korea since May 2023, the user base remains limited, and the follow-up period of less than 1 year is inadequate for a comprehensive analysis. Vericiguat is a promising therapeutic option for HF in dialysis patients. Future clinical data, derived from pharmacokinetic studies in individuals with eGFR < 15 mL/min/1.73 m^2^ or on dialysis, will be crucial for determining its efficacy and safety within this population.

In our study, UFV variability was examined as a continuous variable due to the absence of a universally recognized cutoff value that delineates clinically relevant UFV variability among patients undergoing HD. Previous studies investigating UFV variability have similarly classified it as dichromatic, utilizing median or specific values [2,15]. Our quartile analysis indicates that patients in the highest UFV variability quartile (Q4; mean: 0.77 L, cutoff > 0.60 L) demonstrated a significantly increased risk of all-cause mortality, CVE, and dementia compared to those in the lowest quartile (Q1; mean: 0.22 L, cutoff < 0.31 L). This finding indicates that significant month-to-month fluctuation in UFV (> 0.60 L) may signify unstable volume control and suboptimal fluid management. This fluctuation may indicate recurrent hemodynamic abnormalities during HD sessions, including intradialytic hypotension or post-dialysis volume depletion, and compensatory overhydration between sessions. In clinical practice, when recurrent hemodynamic instability occurs during dialysis, clinicians should first evaluate whether high UFV targets, inaccurate dry weight estimation, or inappropriate dialysis prescriptions are responsible for these fluctuations. The variability of UFV can be easily calculated in routine clinical practice using data from monthly dialysis treatment records, as UFV is routinely measured and documented throughout each session. Regular monitoring of UFV variability may help identify patients with unstable fluid management. Continuous or monthly monitoring of UFV variability can serve as an early warning signal for fluid imbalance, facilitating prompt adjustment of ultrafiltration targets, reassessment of dry weight, or modification of antihypertensive therapy to improve cardiovascular stability and long-term outcomes.

Regrettably, data on intradialytic hypotension, residual renal function, and dialysis modality (e.g., hemodiafiltration versus conventional HD) were unavailable. We recognize that these factors could have influenced patient outcomes by affecting hemodynamic stability, fluid status, and solute clearance. Our dataset comprises the essential data related to the quality improvement of dialysis centers and claims data. Therefore, data about residual renal function or the occurrence of intradialytic hypotension were excluded from our database. Furthermore, in South Korea, despite employing various dialysis modalities, including hemodiafiltration and conventional HD, and different membrane types, a distinctive reimbursement system mechanism ensures uniform coverage regardless of the modality utilized. Therefore, although information on dialysis utilization and frequency is accessible, data on specific modalities or dialysis-related interventions are unavailable in the claims database. However, given the long HD vintage and the low utilization rate of hemodiafiltration in our dataset (approximately > 88% underwent HD only in 2024), it is likely that most patients had minimal or no residual renal function and that nearly all were maintained on conventional HD [24]. Therefore, these factors were unlikely to have significantly influenced. Future studies incorporating these parameters will be essential to further elucidate their independent contributions to outcome variability.

Our study included adult patients undergoing maintenance HD at least twice weekly for ≥ 3 months; however, some patients received dialysis using a central venous catheter. In these cases, some patients may be classified as transitory HD patients, who are more likely to discontinue or interrupt treatment. Additionally, the catheter use may have been transient, either due to vascular access failure from an arteriovenous fistula or graft or while waiting for the maturation of a new access. Furthermore, compared to arteriovenous fistula or graft, catheter use is associated with a higher risk of mechanical complications and infections, potentially hindering effective dialysis administration and ultrafiltration efficiency. Therefore, HD using a catheter could have affected the accuracy of UFV variability measurement and its association with clinical outcomes. To minimize this potential bias and ensure a more stable and homogeneous study population, patients using catheters for vascular access were excluded from the final analysis.

Assuming that UFV variability adversely affects clinical outcomes and that reducing this variability is desirable, the identification of modifiable contributors may be clinically important. In patients without significant residual renal function, two predominant scenarios likely drive UFV instability: (1) excessive volume overload accompanied by frequent insufficient UFV, which results in wide UFV fluctuations, and (2) low UFV resulting from malnutrition, which limits interdialytic fluid intake. In the first scenario, restricting the overall dietary salt and fluid intake could attenuate UFV fluctuations. In the second scenario, strategies to improve nutritional status, such as the use of appetite stimulants or intravenous nutritional supplementation, may help stabilize UFV by restoring physiological fluid gain. Therefore, individualized interventions tailored to the underlying cause of UFV variability may be essential to reduce hemodynamic instability and its potential impacts.

Our study had some limitations. First, it was retrospective, and the baseline characteristics differed significantly among the four groups, likely owing to selection bias. However, the differences between the groups were not substantial, and we aimed to address these limitations through multivariable and subgroup analyses. Second, several important indicators associated with prognosis were not included. In our study, data on the duration of dialysis sessions was unavailable, which prevented us from calculating ultrafiltration rate data. Additionally, data on intradialytic hypotension events, cardiac indices such as echocardiography, endothelial markers, and causes of death were lacking, precluding analysis of cardiovascular mortality. Moreover, data on residual renal function, which could significantly influence ultrafiltration, were also unavailable. Nonetheless, recent Korean registry data indicate that 93% of HD patients undergo dialysis three times per week, with only 9.3% receiving sessions longer than 4.5 h and 2.0% receiving sessions shorter than 3.5 h [24,25]. Thus, the majority of patients likely undergo HD three times per week for an average duration of 4 h per session. Furthermore, the mean HD vintage in our study was approximately 5 years or more. Most patients in our study may have little residual renal function. Third, our study lacks detailed data on HD modalities, which may have served as additional confounding factors affecting patient outcomes. In South Korea, reimbursement for HD sessions is provided as a fixed bundle that uniformly covers all associated costs, including materials, laboratory tests, medications, dialyzers, and personnel expenses, regardless of the type of dialyzer or dialysis modality employed. Therefore, conventional HD is predominantly utilized, whereas medium-cutoff dialyzers and hemodiafiltration remain limited to a small proportion of patients. Population-based registry data provides information about dialysis modalities. In 2018, 17% of patients received hemodiafiltration at least once weekly; however, this proportion declined to 11.1% by 2022, indicating that the overwhelming majority of patients continue to receive conventional HD [24]. Given this distribution, it is unlikely that dialysis modality exerted a significant confounding effect in our study. Fourth, our study did not include important variables associated with cardiovascular outcomes, such as echocardiography. It was conducted using only the data recorded at the time of the HD quality assessment program and the prescription-related claim data of the corresponding patients. We did not conduct a detailed chart review that included comprehensive clinical information. Additionally, we implemented the HD quality assessment program as the primary dataset for this study to ensure nationwide quality control of HD centers. It includes parameters that are evidence-based, cost-effective, and widely accessible in most HD centers [4]. Although echocardiography plays a critical role in the diagnosis and prognostic evaluation of heart disease, its routine implementation in private HD centers is often hindered by resource limitations. Furthermore, the lack of consensus regarding standardized criteria for echocardiography reporting and registration has limited its inclusion in this dataset. Consequently, data pertaining to the indications for echocardiography or the examination results were unavailable for analysis in our study. While it may be possible to assess whether echocardiography was performed, including this variable without data on its indication or findings could introduce significant confounding. Therefore, we chose not to include it as a covariate in our analysis.

In conclusion, our population-based cohort study demonstrated an association between high UFV variability and various clinical outcomes, particularly all-cause mortality and dementia. Overall, the association appeared to be stronger in patients with a high CCI, mean UFV < 3 L/session, or long HD vintage. These findings suggest that UFV variability could serve as a useful supplementary indicator for predicting prognosis, in addition to UFV or ultrafiltration rate. However, due to the various limitations of this study, further research is needed to confirm this association more definitively.

## Supplementary Material

Supplementary materials_clean copy.docx

## Data Availability

Raw data were generated at the Health Insurance Review and Assessment Service. The database can be requested from the Health Insurance Review and Assessment Service by sending a study proposal, including the purpose of the study, study design, and duration of analysis, to the website (https://www.hira.or.kr). The authors cannot distribute the data without permission. Further enquiries can be directed to the corresponding author.
