# Supplementary material for: Association between ultrafiltration variability and clinical outcomes in patients undergoing hemodialysis
Source: Ren Fail. 2026 Feb 11;48(1):2620166. doi: 10.1080/0886022X.2026.2620166 (PMC12903931; doi:10.1080/0886022X.2026.2620166)
Supplement: Supplementary materials_clean copy.docx [file IRNF_A_2620166_SM1848.docx]

**Table S1.** Medication types and Health Insurance Review and Assessment Service codes

**Table S2.** Variability of UFV and HR of all-cause mortality according to subgroup

**Table S3.** Variability of UFV and HR of cardiovascular events according to subgroup

**Table S4.** Variability of UFV and HR of atrial fibrillation according to subgroup

**Table S5.** Variability of UFV and HR of dementia according to subgroup

**Table S6.** Variability of UFV and HR of clinical outcomes according to subgroup of heart failure

**Table S7.** Baseline characteristics according to quartiles of variability in weight-adjusted ultrafiltration volume

**Table S8.** Cox regression analyses according to quartiles of variability in weight-adjusted ultrafiltration volume

**Table S9.** Baseline characteristics of Q1 and Q4 groups after propensity score matching

**Table S10.** Cox regression analyses using propensity score matching

**Figure S1.** Spline curves illustrating hazard ratios and 95% confidence intervals for clinical outcomes according to variability of UFV.

**Figure S2.** Kaplan–Meier curves of patient survival, cardiovascular events, atrial fibrillation, and dementia according to quartiles of variability in weight-adjusted ultrafiltration volume

**Figure S3.** Distribution of propensity scores before and after matching

**Table S1. Medication types and Health Insurance Review and Assessment Service codes**

| **Medications** | **Codes** |
| --- | --- |
| **Alacepril** | 104201ATB, 104202ATB |
| **Benazepril** | 114701ATB |
| **Captopril** | 122901ATB, 122902ATB, 122903ATB |
| **Cilazapril** | 133001ATB, 133002ATB, 133003ATB |
| **Enalapril** | 151601ATB, 151603ATB |
| **Fosinopril** | 163501ATB, 163502ATB |
| **Imidapril** | 173401ATB, 173402ATB |
| **Moexipril** | 196801ATB, 196802ATB |
| **Lisinopril** | 184501ATB |
| **Perindopril** | 211301ATB, 211302ATB, 501601ATB, 501602ATB |
| **Quinapril** | 221901ATB, |
| **Ramipril** | 222401ATB, 222402ATB, 222404ATB |
| **Zofenopril** | 510401ATB, 510402ATB, 510403ATB |
| **Temocapril** | 235002ATB |
| **Delapril** | 140901ATB, 140902ATB |
| **Captopril + hydrochlorothiazide** | 262200ATB, 262300ATB |
| **Enalapril + hydrochlorothiazide** | 440300ATB, 453700ATB, 453600ATB |
| **Ramipril + felodipine** | 447100ATB, 447200ATB |
| **Ramipril + hydrochlorothiazide** | 448600ATB, 448700ATB |
| **Perindopril + indapamide** | 556200ATB |
| **Lisinopril + hydrochlorothiazide** | 499200ATB, 499300ATB |
| **Moexipril + hydrochlorothiazide** | 440800ATB, 497900ATB |
| **Enalapril + nitrendipine** | 466000ATB |
| **Candesartan** | 122601ATB, 122602ATB, 122603ATB |
| **Irbesartan** | 177301ATB, 177303ATB |
| **Losartan** | 185701ATB, 185702ATB |
| **Valsartan** | 247101ATB, 247102ATB, 247103ATB, 247104ATB |
| **Fimasartan** | 515201ATB, 515202ATB, 515203ATB |
| **Azilsartan** | 662401ATB, 662402ATB, 662403ATB |
| **Telmisartan** | 378801ATB, 378802ATB |
| **Eprosartan** | 429201ATB |
| **Olmesartan** | 468501ATB, 468502ATB, 468503ATB, 520901ATB, 520902ATB |
| **Valsartan + amlodipine** | 492800ATB, 492900ATB, 495800ATB, 522600ABTB, 522700ABTB, 522800ABTB, 522900ABTB, 523000ATB, 523100ATB, 523200ATB, 523300ATB, 523400ATB |
| **Valsartan + lercanidipne** | 522200ATB. 522300ATB. 522400ATB |
| **Valsartan + pitavastatin** | 634900ATB, 635000ATB, 635100ATB, 635200ATB |
| **Valsartan + sacubitril** | 651401ATB, 651402ATB, 651403ATB |
| **Valsartan + rosuvastatin** | 629700ATB, 629800ATB, 525000ATB, 525100ATB, 525200ATB, 525300ATB, |
| **Valsartan + hydrochlorothiazide** | 356400ATB, 442600ATB |
| **Olmesaetan + amlodipine** | 500500ATB, 500600ATB, 547500ATB. 547600ATB, 547700ATB, 547800ATB, 547900ATB, 548000ATB, 582200ATB, 582400ATB, 629400ATB, 629500ATB, 629600ATB, 631300ATB, 632800ATB, 632900ATB, 633000ATB |
| **Olmesartan + hydrochlorothiazide** | 513600ATB |
| **Olmesartan + hydrochlorothiazide + amlodipine** | 519700ATB, 519800ATB, 519900ATB, 520000ATB, 520100ATB |
| **Olmesartan + rosuvastatin** | 653200ATB, 644100ATB, 644200ATB, 526300ATB, 526400ATB, 526500ATB, 526900ATB |
| **Telmisartan + hydrochlorothiazide** | 502600ATB, 443200ATB, 443300ATB |
| **Telmisartan + rosuvastatin** | 629900ATB, 630000ATB, 630100ATB, 630200ATB, 631600ATB, 631700ATB |
| **Telmisartan + amlodipine** | 511500ATB, 511600ATB, 511700ATB, 521200ATB, 521300ATB, 521400ATB, 623100ATB, 644800ATB |
| **Telmisartan+ hydrochlorothiazide + amlodipine** | 663500ATB, 663600ATB, 663700ATB, 663800ATB |
| **Telmisartan + rosuvastatin + amlodipine** | 671700ATB, 671600ATB, 671500ATB, 671400ATB, 671300ATB, 671200ATB, |
| **Losartan + hydrochlorothiazide** | 262500ATB, 378900ATB, 486900ATB |
| **Losartan + amlodipine** | 502700ATB, 503000ATB, 513900ATB, 637400ATB, 637500ATB, 637600ATB |
| **Losartan + rosuvastatin+ amlodipine** | 663900ATB, 664000ATB, 664100ATB, 664200ATB, 664300ATB, 664400ATB, |
| **Losartan + hydrochlorothiazide+ amlodipine** | 662800ATB, 662900ATB, 663000ATB |
| **Fimasartan + hydrochlorothiazide** | 522000ATB, 526800ATB |
| **Fimasartan + amlodipine** | 651900ATB, 652000ATB, 652100ATB, 652700ATB, 651900ATB |
| **Fimasartan + rosuvastatin** | 654600ATB, 654700ATB, 654800ATB, 654900ATB, 655000ATB |
| **Candesartan + hydrochlorothiazide** | 423700ATB |
| **Candesartan + amlodipine** | 652900ATB, 653000ATB, 653100ATB, 652900ATB, 652900ATB |
| **Candesartan + rosuvastatin** | 673700ATB, 661800ATB, 661900ATB, 662000ATB, 662100ATB |
| **Irbesartan + hydrochlorothiazide** | 385700ATB, 385800ATB |
| **Irbesartan + atorvastatin** | 527000ATB, 527100ATB, 524000ATB, 524100ATB |
| **Azilsartan + chlorthalidone** | 673500ATB, 673600ATB |
| **Eprosartan + hydrochlorothiazide** | 460500ATB |
| **Amlodipine + atorvastatin** | 614500ATB, 472300ATB, 472400ATB, 472500ATB, 518900ATB |
| **Amlodipine + rosuvastatin** | 673900ATB, 674000ATB, 674100ATB |
| **Atorvastatin + ezetimibe** | 633800ATB, 633900ATB, 634800ATB |
| **Pitavastatin + fenofibrate** | 679300ACH |
| **Rosuvastatin + ezetimibe** | 640700ATB, 640800ATB, 640900ATB |
| **Metformin + atorvastatin** | 671800ATR, 673800ATR, 671900ATR, 672000ATR, 672100ATR |
| **Metformin + rosuvastatin** | 672500ATR, 672600ATR, 672700ATR, 672800ATR, 672900ATR, 673000ATR, 683300ATR, 683400ATR |
| **Gemigliptin + rosuvastatin** | 664600ATB, 664700ATB, 664800ATB |
| **Aspirin** | 110701ATB, 110702ATB, 110801ATB, 110802ATB, 111001ACE, 111001ATB, 111001ATE, 111002ATE, 111003ACE, 111003ATE |
| **Clopidogrel** | 133201ACR, 133201ATB, 133201ATR, 133202ATB, 133203ATR, 506100ATB |
| **Cilostazol** | 136901ATB, 492501ATB, 495201ATB, 498801ATB, 501501ATB |
| **Ticlopidine** | 498900ATB, 239201ATB, 239202ATB |
| **Aspirin + bethocarbamol** | 256800ATB |
| **Aspirin + clopidogrel** | 517900ACH, 517900ACE, 517900ATE, 667500ACE |
| **Aspirin + dipyridamole** | 489700ACR |
| **Atorvastatin** | 111502ATB, 502202ATB, 633900ATB, 472400ATB, 518900ATB, 524100ATB, 527000ATB, 672000ATR, 672100ATR, 111503ATB, 502203ATB, 634800ATB, 472500ATB, 111504ATB, 502204ATB |
| **Fluvastatin** | 162401ACH, 162402ACH, 162403ATR |
| **Lovastatin** | 185801ATB |
| **Pitavastatin** | 470901ATB, 470902ATB, 470903ATB |
| **Pravastatin** | 216601ATB, 216602ATB, 216603ATB, 216604ATB |
| **Rosuvastatin** | 454001ATB, 454002ATD, 454002ATB, 454003ATB, 454003ATD, 454005ATB |
| **Simvastatin** | 227801ATB, 227802ATB, 227803ATB, 227805ATB, 227806ATB |

**Table S2. Variability of UFV and HR of all-cause mortality according to subgroup**

|  | **Multivariable** | |  | | **Multivariable** | | ***P* for interaction** |
| --- | --- | --- | --- | --- | --- | --- | --- |
|  | **Mean (95% CI)** | ***P*** | |  | **Mean (95% CI)** | ***P*** |  |
| **Males** |  |  | | **Females** |  |  | 0.060 |
| Ref: Q1 |  |  | |  |  |  |  |
| Q2 | 0.99 (0.93–1.05) | 0.719 | |  | 1.01 (0.94–1.09) | 0.759 |  |
| Q3 | 1.00 (0.94–1.06) | 0.994 | |  | 1.07 (0.99–1.16) | 0.085 |  |
| Q4 | 1.08 (1.02–1.15) | 0.013 | |  | 1.16 (1.07–1.26) | <0.001 |  |
| Ref: Q2 |  |  | |  |  |  |  |
| Q3 | 1.01 (0.95–1.08) | 0.713 | |  | 1.06 (0.98–1.14) | 0.151 |  |
| Q4 | 1.09 (1.03–1.16) | 0.003 | |  | 1.15 (1.06–1.25) | <0.001 |  |
| Ref: Q3 |  |  | |  |  |  |  |
| Q4 | 1.08 (1.02–1.14) | 0.008 | |  | 1.09 (1.01–1.18) | 0.043 |  |
| **< 65 years old** |  |  | | **≥ 65** |  |  | 0.181 |
| Ref: Q1 |  |  | |  |  |  |  |
| Q2 | 1.00 (0.91–1.09) | 0.991 | |  | 1.00 (0.94–1.06) | 0.945 |  |
| Q3 | 1.05 (0.96–1.14) | 0.303 | |  | 1.02 (0.97–1.09) | 0.431 |  |
| Q4 | 1.12 (1.03–1.22) | 0.010 | |  | 1.11 (1.04–1.18) | <0.001 |  |
| Ref: Q2 |  |  | |  |  |  |  |
| Q3 | 1.05 (0.96–1.14) | 0.298 | |  | 1.03 (0.97–1.09) | 0.382 |  |
| Q4 | 1.12 (1.03–1.21) | 0.009 | |  | 1.11 (1.05–1.18) | <0.001 |  |
| Ref: Q3 |  |  | |  |  |  |  |
| Q4 | 1.07 (0.99–1.16) | 0.111 | |  | 1.08 (1.02–1.15) | 0.007 |  |
| **HDV < 41 months** |  |  | | **HDV ≥ 41** |  |  | 0.591 |
| Ref: Q1 |  |  | |  |  |  |  |
| Q2 | 0.95 (0.89–1.02) | 0.194 | |  | 1.04 (0.97–1.11) | 0.256 |  |
| Q3 | 1.02 (0.95–1.09) | 0.656 | |  | 1.04 (0.98–1.11) | 0.210 |  |
| Q4 | 1.11 (1.03–1.19) | 0.005 | |  | 1.12 (1.40–1.19) | 0.001 |  |
| Ref: Q2 |  |  | |  |  |  |  |
| Q3 | 1.07 (0.99–1.15) | 0.079 | |  | 1.00 (0.94–1.07) | 0.910 |  |
| Q4 | 1.16 (1.08–1.25) | <0.001 | |  | 1.07 (1.01–1.14) | 0.029 |  |
| Ref: Q3 |  |  | |  |  |  |  |
| Q4 | 1.10 (1.02–1.17) | 0.015 | |  | 1.07 (1.00–1.14) | 0.036 |  |
| **CCI < 7** |  |  | | **CCI ≥ 7** |  |  | 0.576 |
| Ref: Q1 |  |  | |  |  |  |  |
| Q2 | 1.08 (0.93–1.25) | 0.308 | |  | 0.99 (0.94–1.04) | 0.737 |  |
| Q3 | 1.04 (0.90–1.21) | 0.561 | |  | 1.03 (0.98–1.09) | 0.251 |  |
| Q4 | 1.16 (0.99–1.34) | 0.055 | |  | 1.11 (1.05–1.17) | <0.001 |  |
| Ref: Q2 |  |  | |  |  |  |  |
| Q3 | 0.97 (0.84–1.12) | 0.662 | |  | 1.04 (0.99–1.09) | 0.130 |  |
| Q4 | 1.07 (0.93–1.24) | 0.345 | |  | 1.12 (1.06–1.18) | <0.001 |  |
| Ref: Q3 |  |  | |  |  |  |  |
| Q4 | 1.11 (0.96–1.28) | 0.170 | |  | 1.08 (1.02–1.13) | 0.004 |  |
| **UFV ≤ 1 L/session** |  |  | | **1 < UFV ≤ 2** |  |  | 0.262 |
| Ref: Q1 |  |  | |  |  |  |  |
| Q2 | 0.97 (0.83–1.14) | 0.738 | |  | 1.02 (0.94–1.12) | 0.594 |  |
| Q3 | 1.14 (0.95–1.36) | 0.149 | |  | 1.04 (0.95–1.14) | 0.373 |  |
| Q4 | 1.27 (1.05–1.53) | 0.014 | |  | 1.12 (1.03–1.23) | 0.010 |  |
| Ref: Q2 |  |  | |  |  |  |  |
| Q3 | 1.17 (0.97–1.42) | 0.097 | |  | 1.02 (0.94–1.10) | 0.708 |  |
| Q4 | 1.30 (1.07–1.59) | 0.009 | |  | 1.10 (1.01–1.19) | 0.031 |  |
| Ref: Q3 |  |  | |  |  |  |  |
| Q4 | 1.11 (0.90–1.37) | 0.315 | |  | 1.08 (0.99–1.17) | 0.068 |  |
| **2< UFV ≤ 3** |  |  | | **3 < UFV** |  |  |  |
| Ref: Q1 |  |  | |  |  |  |  |
| Q2 | 1.01 (0.93–1.09) | 0.841 | |  | 0.98 (0.87–1.10) | 0.721 |  |
| Q3 | 1.06 (0.98–1.14) | 0.137 | |  | 0.98 (0.87–1.10) | 0.700 |  |
| Q4 | 1.13 (1.05–1.22) | 0.001 | |  | 1.08 (0.96–1.21) | 0.208 |  |
| Ref: Q2 |  |  | |  |  |  |  |
| Q3 | 1.05 (0.98–1.13) | 0.177 | |  | 1.00 (0.88–1.13) | 0.978 |  |
| Q4 | 1.13 (1.05–1.21) | 0.001 | |  | 1.10 (0.98–1.24) | 0.118 |  |
| Ref: Q3 |  |  | |  |  |  |  |
| Q4 | 1.07 (0.99–1.15) | 0.051 | |  | 1.10 (0.98–1.25) | 0.111 |  |

Multivariable analysis was adjusted for age, sex, body mass index, vascular access type, diabetes, HDV, CCI, UF, Kt/V_urea_, levels of hemoglobin, serum albumin, serum creatinine, serum phosphorus, and serum calcium, use of renin–angiotensin system blockers, statins, clopidogrel, aspirin, or anti-hypertensive drugs, presence of myocardial infarction or congestive heart failure, and atrial fibrillation.

**Abbreviations**: CCI, Charlson Comorbidity Index; CI, confidence interval; HDV, hemodialysis vintage; HR, hazard ratio; UFV, ultrafiltration volume.

**Table S3. Variability of UFV and HR of cardiovascular events according to subgroup**

|  | **Multivariable** | |  | | **Multivariable** | | ***P* for interaction** |
| --- | --- | --- | --- | --- | --- | --- | --- |
|  | **Mean (95% CI)** | ***P*** | |  | **Mean (95% CI)** | ***P*** |  |
| **Males** |  |  | | **Females** |  |  | 0.929 |
| Ref: Q1 |  |  | |  |  |  |  |
| Q2 | 1.08 (0.97–1.20) | 0.170 | |  | 1.01 (0.90–1.15) | 0.820 |  |
| Q3 | 1.09 (0.98–1.21) | 0.130 | |  | 1.05 (0.92–1.19) | 0.456 |  |
| Q4 | 1.15 (1.04–1.28) | 0.008 | |  | 1.13 (0.99–1.29) | 0.076 |  |
| Ref: Q2 |  |  | |  |  |  |  |
| Q3 | 1.01 (0.91–1.11) | 0.911 | |  | 1.03 (0.91–1.17) | 0.599 |  |
| Q4 | 1.07 (0.96–1.18) | 0.210 | |  | 1.11 (0.97–1.27) | 0.117 |  |
| Ref: Q3 |  |  | |  |  |  |  |
| Q4 | 1.06 (0.96–1.17) | 0.240 | |  | 1.07 (0.94–1.23) | 0.296 |  |
| **< 65 years old** |  |  | | **≥ 65** |  |  | 0.453 |
| Ref: Q1 |  |  | |  |  |  |  |
| Q2 | 0.94 (0.84–1.06) | 0.336 | |  | 1.16 (1.03–1.30) | 0.012 |  |
| Q3 | 1.08 (0.97–1.21) | 0.166 | |  | 1.06 (0.94–1.19) | 0.338 |  |
| Q4 | 1.11 (0.99–1.25) | 0.060 | |  | 1.17 (1.04–1.32) | 0.009 |  |
| Ref: Q2 |  |  | |  |  |  |  |
| Q3 | 1.15 (1.02–1.29) | 0.017 | |  | 0.91 (0.82–1.02) | 0.118 |  |
| Q4 | 1.18 (1.06–1.32) | 0.004 | |  | 1.01 (0.91–1.14) | 0.832 |  |
| Ref: Q3 |  |  | |  |  |  |  |
| Q4 | 1.03 (0.92–1.14) | 0.616 | |  | 1.11 (0.99–1.24) | 0.086 |  |
| **HDV < 41 months** |  |  | | **HDV ≥ 41** |  |  | 0.590 |
| Ref: Q1 |  |  | |  |  |  |  |
| Q2 | 0.98 (0.87–1.11) | 0.771 | |  | 1.12 (0.99–1.25) | 0.055 |  |
| Q3 | 1.00 (0.89–1.13) | 0.960 | |  | 1.13 (1.01–1.27) | 0.033 |  |
| Q4 | 1.10 (0.98–1.24) | 0.102 | |  | 1.17 (1.04–1.31) | 0.008 |  |
| Ref: Q2 |  |  | |  |  |  |  |
| Q3 | 1.02 (0.91–1.15) | 0.735 | |  | 1.01 (0.91–1.13) | 0.830 |  |
| Q4 | 1.12 (0.99–1.26) | 0.055 | |  | 1.04 (0.94–1.16) | 0.449 |  |
| Ref: Q3 |  |  | |  |  |  |  |
| Q4 | 1.10 (0.98–1.24) | 0.112 | |  | 1.03 (0.93–1.15) | 0.581 |  |
| **CCI < 7** |  |  | | **CCI ≥ 7** |  |  | 0.172 |
| Ref: Q1 |  |  | |  |  |  |  |
| Q2 | 1.11 (0.92–1.35) | 0.279 | |  | 1.04 (0.95–1.14) | 0.374 |  |
| Q3 | 1.25 (1.03–1.52) | 0.022 | |  | 1.04 (0.95–1.13) | 0.434 |  |
| Q4 | 1.23 (1.01–1.50) | 0.040 | |  | 1.12 (1.02–1.22) | 0.014 |  |
| Ref: Q2 |  |  | |  |  |  |  |
| Q3 | 1.12 (0.93–1.35) | 0.216 | |  | 1.00 (0.91–1.09) | 0.913 |  |
| Q4 | 1.10 (0.91–1.33) | 0.311 | |  | 1.07 (0.98–1.17) | 0.110 |  |
| Ref: Q3 |  |  | |  |  |  |  |
| Q4 | 0.98 (0.82–1.18) | 0.845 | |  | 1.08 (0.99–1.18) | 0.084 |  |
| **UFV ≤ 1 L/session** |  |  | | **1 < UFV ≤ 2** |  |  | 0.759 |
| Ref: Q1 |  |  | |  |  |  |  |
| Q2 | 1.27 (0.97–1.67) | 0.086 | |  | 1.11 (0.95–1.30) | 0.190 |  |
| Q3 | 0.99 (0.71–1.38) | 0.961 | |  | 1.19 (1.02–1.39) | 0.029 |  |
| Q4 | 1.31 (0.94–1.83) | 0.114 | |  | 1.24 (1.06–1.45) | 0.007 |  |
| Ref: Q2 |  |  | |  |  |  |  |
| Q3 | 0.78 (0.56–1.09) | 0.147 | |  | 1.07 (0.93–1.24) | 0.362 |  |
| Q4 | 1.03 (0.73–1.45) | 0.855 | |  | 1.12 (0.96–1.29) | 0.144 |  |
| Ref: Q3 |  |  | |  |  |  |  |
| Q4 | 1.32 (0.90–1.94) | 0.153 | |  | 1.04 (0.90–1.20) | 0.567 |  |
| **2< UFV ≤ 3** |  |  | | **3 < UFV** |  |  |  |
| Ref: Q1 |  |  | |  |  |  |  |
| Q2 | 0.99 (0.88–1.13) | 0.931 | |  | 1.06 (0.89–1.26) | 0.504 |  |
| Q3 | 1.02 (0.90–1.16) | 0.751 | |  | 1.06 (0.89–1.27) | 0.496 |  |
| Q4 | 1.06 (0.93–1.20) | 0.370 | |  | 1.20 (1.01–1.43) | 0.039 |  |
| Ref: Q2 |  |  | |  |  |  |  |
| Q3 | 1.03 (0.91–1.16) | 0.672 | |  | 1.00 (0.84–1.20) | 0.984 |  |
| Q4 | 1.07 (0.94–1.20) | 0.304 | |  | 1.13 (0.95–1.35) | 0.173 |  |
| Ref: Q3 |  |  | |  |  |  |  |
| Q4 | 1.04 (0.92–1.17) | 0.530 | |  | 1.13 (0.94–1.35) | 0.183 |  |

Multivariable analysis was adjusted for age, sex, body mass index, vascular access type, diabetes, HDV, CCI, UF, Kt/V_urea_, levels of hemoglobin, serum albumin, serum creatinine, serum phosphorus, and serum calcium, use of renin–angiotensin system blockers, statins, clopidogrel, aspirin, or anti-hypertensive drugs, presence of myocardial infarction or congestive heart failure, and atrial fibrillation.

**Abbreviations**: CCI, Charlson Comorbidity Index; CI, confidence interval; HDV, hemodialysis vintage; HR, hazard ratio; UFV, ultrafiltration volume.

**Table S4. Variability of UFV and HR of atrial fibrillation according to subgroup**

|  | **Multivariable** | |  | | **Multivariable** | | ***P* for interaction** |
| --- | --- | --- | --- | --- | --- | --- | --- |
|  | **Mean (95% CI)** | ***P*** | |  | **Mean (95% CI)** | ***P*** |  |
| **Males** |  |  | | **Females** |  |  | 0.498 |
| Ref: Q1 |  |  | |  |  |  |  |
| Q2 | 1.01 (0.90–1.13) | 0.880 | |  | 0.98 (0.86–1.12) | 0.812 |  |
| Q3 | 1.00 (0.90–1.12) | 0.967 | |  | 1.09 (0.96–1.25) | 0.194 |  |
| Q4 | 1.08 (0.97–1.20) | 0.155 | |  | 1.13 (0.98–1.30) | 0.086 |  |
| Ref: Q2 |  |  | |  |  |  |  |
| Q3 | 0.99 (0.89–1.10) | 0.909 | |  | 1.11 (0.97–1.27) | 0.127 |  |
| Q4 | 1.07 (0.97–1.19) | 0.195 | |  | 1.15 (0.99–1.33) | 0.053 |  |
| Ref: Q3 |  |  | |  |  |  |  |
| Q4 | 1.08 (0.97–1.19) | 0.147 | |  | 1.04 (0.90–1.19) | 0.622 |  |
| **< 65 years old** |  |  | | **≥ 65** |  |  | 0.846 |
| Ref: Q1 |  |  | |  |  |  |  |
| Q2 | 0.96 (0.85–1.09) | 0.553 | |  | 1.02 (0.91–1.14) | 0.756 |  |
| Q3 | 0.97 (0.85–1.10) | 0.600 | |  | 1.08 (0.97–1.21) | 0.170 |  |
| Q4 | 1.08 (0.96–1.22) | 0.204 | |  | 1.10 (0.98–1.23) | 0.113 |  |
| Ref: Q2 |  |  | |  |  |  |  |
| Q3 | 1.00 (0.89–1.14) | 0.939 | |  | 1.06 (0.95–1.19) | 0.281 |  |
| Q4 | 1.12 (0.99–1.27) | 0.056 | |  | 1.08 (0.96–1.21) | 0.192 |  |
| Ref: Q3 |  |  | |  |  |  |  |
| Q4 | 1.12 (0.99–1.26) | 0.062 | |  | 1.02 (0.91–1.14) | 0.792 |  |
| **HDV < 41 months** |  |  | | **HDV ≥ 41** |  |  | 0.672 |
| Ref: Q1 |  |  | |  |  |  |  |
| Q2 | 0.98 (0.86–1.11) | 0.741 | |  | 1.02 (0.91–1.14) | 0.740 |  |
| Q3 | 1.04 (0.92–1.18) | 0.526 | |  | 1.04 (0.92–1.16) | 0.548 |  |
| Q4 | 1.06 (0.93–1.20) | 0.396 | |  | 1.13 (1.01–1.27) | 0.031 |  |
| Ref: Q2 |  |  | |  |  |  |  |
| Q3 | 1.06 (0.94–1.21) | 0.337 | |  | 1.02 (0.91–1.13) | 0.785 |  |
| Q4 | 1.08 (0.95–1.22) | 0.240 | |  | 1.11 (0.99–1.24) | 0.060 |  |
| Ref: Q3 |  |  | |  |  |  |  |
| Q4 | 1.01 (0.90–1.15) | 0.826 | |  | 1.09 (0.98–1.22) | 0.103 |  |
| **CCI < 7** |  |  | | **CCI ≥ 7** |  |  | 0.227 |
| Ref: Q1 |  |  | |  |  |  |  |
| Q2 | 0.92 (0.75–1.12) | 0.404 | |  | 1.02 (0.93–1.12) | 0.689 |  |
| Q3 | 1.06 (0.88–1.29) | 0.530 | |  | 1.03 (0.94–1.13) | 0.535 |  |
| Q4 | 1.18 (0.97–1.44) | 0.089 | |  | 1.08 (0.99–1.19) | 0.091 |  |
| Ref: Q2 |  |  | |  |  |  |  |
| Q3 | 1.16 (0.95–1.41) | 0.140 | |  | 1.01 (0.92–1.11) | 0.856 |  |
| Q4 | 1.29 (1.06–1.56) | 0.011 | |  | 1.06 (0.97–1.16) | 0.191 |  |
| Ref: Q3 |  |  | |  |  |  |  |
| Q4 | 1.11 (0.92–1.34) | 0.271 | |  | 1.05 (0.96–1.15) | 0.271 |  |
| **UFV ≤ 1 L/session** |  |  | | **1 < UFV ≤ 2** |  |  | 0.905 |
| Ref: Q1 |  |  | |  |  |  |  |
| Q2 | 1.13 (0.86–1.48) | 0.390 | |  | 1.01 (0.85–1.19) | 0.932 |  |
| Q3 | 1.12 (0.82–1.52) | 0.478 | |  | 1.12 (0.96–1.31) | 0.160 |  |
| Q4 | 1.10 (0.78–1.55) | 0.595 | |  | 1.11 (0.95–1.31) | 0.196 |  |
| Ref: Q2 |  |  | |  |  |  |  |
| Q3 | 0.99 (0.72–1.37) | 0.964 | |  | 1.11 (0.96–1.30) | 0.168 |  |
| Q4 | 0.97 (0.68–1.39) | 0.886 | |  | 1.11 (0.95–1.29) | 0.206 |  |
| Ref: Q3 |  |  | |  |  |  |  |
| Q4 | 0.98 (0.67–1.43) | 0.924 | |  | 0.99 (0.86–1.15) | 0.928 |  |
| **2< UFV ≤ 3** |  |  | | **3 < UFV** |  |  |  |
| Ref: Q1 |  |  | |  |  |  |  |
| Q2 | 1.01 (0.88–1.15) | 0.928 | |  | 0.99 (0.82–1.19) | 0.885 |  |
| Q3 | 1.06 (0.93–1.20) | 0.410 | |  | 0.92 (0.76–1.11) | 0.399 |  |
| Q4 | 1.13 (0.99–1.29) | 0.068 | |  | 1.12 (0.93–1.34) | 0.226 |  |
| Ref: Q2 |  |  | |  |  |  |  |
| Q3 | 1.05 (0.93–1.19) | 0.444 | |  | 0.93 (0.77–1.14) | 0.499 |  |
| Q4 | 1.12 (0.99–1.27) | 0.068 | |  | 1.13 (0.94–1.37) | 0.192 |  |
| Ref: Q3 |  |  | |  |  |  |  |
| Q4 | 1.07 (0.95–1.20) | 0.278 | |  | 1.21 (1.00–1.47) | 0.049 |  |

Multivariable analysis was adjusted for age, sex, body mass index, vascular access type, diabetes, HDV, CCI, UF, Kt/V_urea_, levels of hemoglobin, serum albumin, serum creatinine, serum phosphorus, and serum calcium, use of renin–angiotensin system blockers, statins, clopidogrel, aspirin, or anti-hypertensive drugs, and presence of myocardial infarction or congestive heart failure.

**Abbreviations**: CCI, Charlson Comorbidity Index; CI, confidence interval; HDV, hemodialysis vintage; HR, hazard ratio; UFV, ultrafiltration volume.

**Table S5. Variability of UFV and HR of dementia according to subgroup**

|  | **Multivariable** | |  | **Multivariable** | | ***P* for interaction** |
| --- | --- | --- | --- | --- | --- | --- |
|  | **Mean (95% CI)** | ***P*** |  | **Mean (95% CI)** | ***P*** |  |
| **Males** |  |  | **Females** |  |  | 0.456 |
| Ref: Q1 |  |  |  |  |  |  |
| Q2 | 1.23 (1.05–1.43) | 0.008 |  | 1.18 (1.00–1.38) | 0.044 |  |
| Q3 | 1.22 (1.05–1.42) | 0.011 |  | 1.27 (1.08–1.49) | 0.003 |  |
| Q4 | 1.33 (1.15–1.54) | <0.001 |  | 1.40 (1.18–1.65) | <0.001 |  |
| Ref: Q2 |  |  |  |  |  |  |
| Q3 | 0.99 (0.86–1.14) | 0.903 |  | 1.08 (0.93–1.26) | 0.329 |  |
| Q4 | 1.08 (0.95–1.24) | 0.253 |  | 1.19 (1.01–1.39) | 0.036 |  |
| Ref: Q3 |  |  |  |  |  |  |
| Q4 | 1.09 (0.96–1.25) | 0.196 |  | 1.10 (0.94–1.29) | 0.248 |  |
| **< 65 years old** |  |  | **≥ 65** |  |  | 0.525 |
| Ref: Q1 |  |  |  |  |  |  |
| Q2 | 1.31 (1.09–1.59) | 0.005 |  | 1.15 (1.00–1.31) | 0.049 |  |
| Q3 | 1.17 (0.97–1.42) | 0.105 |  | 1.28 (1.12–1.46) | <0.001 |  |
| Q4 | 1.38 (1.14–1.66) | <0.001 |  | 1.35 (1.18–1.55) | <0.001 |  |
| Ref: Q2 |  |  |  |  |  |  |
| Q3 | 0.89 (0.75–1.07) | 0.212 |  | 1.11 (0.98–1.27) | 0.095 |  |
| Q4 | 1.05 (0.88–1.24) | 0.608 |  | 1.18 (1.04–1.34) | 0.013 |  |
| Ref: Q3 |  |  |  |  |  |  |
| Q4 | 1.17 (0.98–1.39) | 0.074 |  | 1.06 (0.93–1.20) | 0.383 |  |
| **HDV < 41 months** |  |  | **HDV ≥ 41** |  |  | 0.029 |
| Ref: Q1 |  |  |  |  |  |  |
| Q2 | 1.08 (0.92–1.25) | 0.346 |  | 1.37 (1.17–1.61) | <0.001 |  |
| Q3 | 1.16 (0.99–1.34) | 0.060 |  | 1.36 (1.16–1.60) | <0.001 |  |
| Q4 | 1.14 (0.98–1.33) | 0.095 |  | 1.62 (1.38–1.90) | <0.001 |  |
| Ref: Q2 |  |  |  |  |  |  |
| Q3 | 1.07 (0.93–1.25) | 0.342 |  | 0.99 (0.86–1.15) | 0.938 |  |
| Q4 | 1.06 (0.91–1.23) | 0.449 |  | 1.19 (1.03–1.37) | 0.019 |  |
| Ref: Q3 |  |  |  |  |  |  |
| Q4 | 0.99 (0.85–1.15) | 0.860 |  | 1.19 (1.03–1.37) | 0.015 |  |
| **CCI < 7** |  |  | **CCI ≥ 7** |  |  | 0.119 |
| Ref: Q1 |  |  |  |  |  |  |
| Q2 | 1.44 (1.07–1.93) | 0.015 |  | 1.18 (1.04–1.32) | 0.007 |  |
| Q3 | 1.22 (0.89–1.67) | 0.212 |  | 1.25 (1.11–140) | <0.001 |  |
| Q4 | 1.22 (0.88–1.68) | 0.234 |  | 1.38 (1.23–1.55) | <0.001 |  |
| Ref: Q2 |  |  |  |  |  |  |
| Q3 | 0.85 (0.64–1.13) | 0.258 |  | 1.06 (0.95–1.19) | 0.289 |  |
| Q4 | 0.85 (0.63–1.14) | 0.272 |  | 1.17 (1.05–1.31) | 0.005 |  |
| Ref: Q3 |  |  |  |  |  |  |
| Q4 | 1.00 (0.73–1.37) | 0.989 |  | 1.10 (0.99–1.23) | 0.075 |  |
| **UFV ≤ 1 L/session** |  |  | **1 < UFV ≤ 2** |  |  | 0.720 |
| Ref: Q1 |  |  |  |  |  |  |
| Q2 | 1.30 (0.93–1.81) | 0.129 |  | 1.18 (0.97–1.44) | 0.097 |  |
| Q3 | 1.13 (0.77–1.66) | 0.535 |  | 1.36 (1.12–1.65) | 0.002 |  |
| Q4 | 1.11 (0.72–1.72) | 0.639 |  | 1.37 (1.13–1.67) | 0.002 |  |
| Ref: Q2 |  |  |  |  |  |  |
| Q3 | 0.87 (0.59–1.28) | 0.483 |  | 1.15 (0.96–1.37) | 0.126 |  |
| Q4 | 0.86 (0.56–1.32) | 0.486 |  | 1.16 (0.97–1.39) | 0.102 |  |
| Ref: Q3 |  |  |  |  |  |  |
| Q4 | 0.98 (0.62–1.57) | 0.943 |  | 1.01 (0.85–1.20) | 0.889 |  |
| **2< UFV ≤ 3** |  |  | **3 < UFV** |  |  |  |
| Ref: Q1 |  |  |  |  |  |  |
| Q2 | 1.21 (1.02–1.43) | 0.030 |  | 1.12 (0.85–1.48) | 0.433 |  |
| Q3 | 1.20 (1.01–1.42) | 0.038 |  | 1.08 (0.81–1.43) | 0.616 |  |
| Q4 | 1.30 (1.10–1.54) | 0.002 |  | 1.51 (1.15–1.97) | 0.003 |  |
| Ref: Q2 |  |  |  |  |  |  |
| Q3 | 0.99 (0.85–1.16) | 0.914 |  | 0.96 (0.72–1.28) | 0.792 |  |
| Q4 | 1.08 (0.92–1.26) | 0.347 |  | 1.35 (1.03–1.76) | 0.031 |  |
| Ref: Q3 |  |  |  |  |  |  |
| Q4 | 1.09 (0.93–1.27) | 0.286 |  | 1.40 (1.06–1.84) | 0.017 |  |

Multivariable analysis was adjusted for age, sex, body mass index, vascular access type, diabetes, HDV, CCI, UF, Kt/V_urea_, levels of hemoglobin, serum albumin, serum creatinine, serum phosphorus, and serum calcium, use of renin–angiotensin system blockers, statins, clopidogrel, aspirin, or anti-hypertensive drugs, presence of myocardial infarction or congestive heart failure, and atrial fibrillation.

**Abbreviations**: CCI, Charlson Comorbidity Index; CI, confidence interval; HDV, hemodialysis vintage; HR, hazard ratio; UFV, ultrafiltration volume.

**Table S6. Variability of UFV and HR of clinical outcomes according to subgroup of heart failure**

|  | **Multivariable** | |  | **Multivariable** | | ***P* for interaction** |
| --- | --- | --- | --- | --- | --- | --- |
|  | **Mean (95% CI)** | ***P*** |  | **Mean (95% CI)** | ***P*** |  |
| **All-cause mortality** | | |  |  |  | 0.116 |
| **Right side** |  |  | **Left side** |  |  |  |
| Ref: Q1 |  |  |  |  |  |  |
| Q2 | 1.00 (0.85–1.18) | 0.999 |  | 1.23 (0.80–1.88) | 0.352 |  |
| Q3 | 0.97 (0.83–1.14) | 0.728 |  | 1.36 (0.92–2.01) | 0.123 |  |
| Q4 | 1.06 (0.90–1.25) | 0.492 |  | 1.70 (1.11–2.60) | 0.014 |  |
| Ref: Q2 |  |  |  |  |  |  |
| Q3 | 0.97 (0.83–1.14) | 0.724 |  | 1.11 (0.74–1.65) | 0.608 |  |
| Q4 | 1.06 (0.90–1.25) | 0.488 |  | 1.39 (0.90–2.15) | 0.143 |  |
| Ref: Q3 |  |  |  |  |  |  |
| Q4 | 1.09 (0.93–1.28) | 0.293 |  | 1.25 (0.84–1.86) | 0.272 |  |
| **Cardiovascular events** | | |  |  |  | 0.538 |
| **Right side** |  |  | **Left side** |  |  |  |
| Ref: Q1 |  |  |  |  |  |  |
| Q2 | 1.05 (0.77–1.44) | 0.749 |  | 0.74 (0.24–2.36) | 0.616 |  |
| Q3 | 0.94 (0.68–1.30) | 0.713 |  | 1.28 (0.51–3.19) | 0.597 |  |
| Q4 | 1.02 (0.74–1.41) | 0.890 |  | 1.20 (0.45–3.20) | 0.714 |  |
| Ref: Q2 |  |  |  |  |  |  |
| Q3 | 0.89 (0.66–1.22) | 0.479 |  | 1.72 (0.59–5.03) | 0.323 |  |
| Q4 | 0.97 (0.71–1.33) | 0.857 |  | 1.61 (0.52–5.01) | 0.408 |  |
| Ref: Q3 |  |  |  |  |  |  |
| Q4 | 1.09 (0.79–1.49) | 0.608 |  | 0.94 (0.38–2.33) | 0.891 |  |
| **Atrial fibrillation** | | |  |  |  | 0.897 |
| **Right side** |  |  | **Left side** |  |  |  |
| Ref: Q1 |  |  |  |  |  |  |
| Q2 | 1.02 (0.76–1.38) | 0.902 |  | 2.06 (0.92–4.59) | 0.078 |  |
| Q3 | 0.89 (0.66–1.20) | 0.448 |  | 0.86 (0.38–1.93) | 0.712 |  |
| Q4 | 0.88 (0.65–1.21) | 0.434 |  | 0.97 (0.41–2.25) | 0.935 |  |
| Ref: Q2 |  |  |  |  |  |  |
| Q3 | 0.87 (0.65–1.18) | 0.377 |  | 0.42 (0.19–0.91) | 0.029 |  |
| Q4 | 0.87 (0.64–1.18) | 0.369 |  | 0.47 (0.20–1.10) | 0.082 |  |
| Ref: Q3 |  |  |  |  |  |  |
| Q4 | 0.99 (0.73–1.36) | 0.964 |  | 1.12 (0.48–2.61) | 0.785 |  |
| **Dementia** | | |  |  |  | 0.806 |
| **Right side** |  |  | **Left side** |  |  |  |
| Ref: Q1 |  |  |  |  |  |  |
| Q2 | 1.29 (0.86–1.94) | 0.220 |  | 1.66 (0.48–5.79) | 0.427 |  |
| Q3 | 1.10 (0.73–1.66) | 0.644 |  | 2.00 (0.64–6.23) | 0.234 |  |
| Q4 | 1.33 (0.87–2.02) | 0.183 |  | 1.37 (0.36–5.19) | 0.646 |  |
| Ref: Q2 |  |  |  |  |  |  |
| Q3 | 0.85 (0.58–1.25) | 0.418 |  | 1.20 (0.42–3.41) | 0.729 |  |
| Q4 | 1.03 (0.70–1.52) | 0.881 |  | 0.82 (0.24–2.88) | 0.762 |  |
| Ref: Q3 |  |  |  |  |  |  |
| Q4 | 1.21 (0.81–1.79) | 0.351 |  | 0.69 (0.22–2.15) | 0.518 |  |

Multivariable analysis was adjusted for age, sex, body mass index, vascular access type, diabetes, hemodialysis vintage, Charlson Comorbidity Index, UFV, Kt/V_urea_, levels of hemoglobin, serum albumin, serum creatinine, serum phosphorus, serum calcium, use of renin–angiotensin system blockers, statins, clopidogrel, aspirin, anti-hypertensive drugs, and presence of atrial fibrillation.

**Abbreviations**: CI, confidence interval; HR, hazard ratio; UFV, ultrafiltration volume.

**Table S7. Baseline characteristics according to quartiles of variability in weight-adjusted ultrafiltration volume.**

|  | **aQ1** | **aQ2** | **aQ3** | **aQ4** | ***P*-value** |
| --- | --- | --- | --- | --- | --- |
| Age (years) | 61.3 ± 12.9 | 62.1 ± 12.7^a^ | 62.2 ± 12.8^a^ | 62.0 ± 13.1^a^ | <0.001 |
| Sex (male, %) | 8,190 (64.8) | 7,751 (61.3) | 7,550 (59.7) | 7,072 (55.9) | <0.001 |
| Hemodialysis vintage (months) | 59 ± 63 | 65 ± 67^a^ | 70 ± 70^ab^ | 73 ± 72^abc^ | <0.001 |
| Body mass index (kg/m^2^) | 24.1 ± 4.0 | 23.1 ± 3.5^a^ | 22.4 ± 3.3^ab^ | 21.4 ± 3.0^abc^ | <0.001 |
| Diabetes (%) | 5,765 (45.6) | 5,622 (44.5) | 5,433 (43.0) | 5,322 (42.1) | <0.001 |
| CCI score | 8.8 ± 2.8 | 8.8 ± 2.9 | 8.8 ± 2.9 | 8.9 ± 2.9 | 0.788 |
| Arteriovenous fistula (%) | 11,083 (87.7) | 10,939 (86.5) | 10,873 (86.0) | 10,718 (84.8) | <0.001 |
| Kt/V_urea_ | 1.52 ± 0.26 | 1.56 ± 0.27^a^ | 1.58 ± 0.27^ab^ | 1.63 ± 0.28^abc^ | <0.001 |
| Ultrafiltration volume (L/session) | 2.43 ± 1.04 | 2.33 ± 0.86^a^ | 2.27 ± 0.79^ab^ | 2.22 ± 0.74^abc^ | <0.001 |
| Hemoglobin (g/dL) | 10.7 ± 0.7 | 10.7 ± 0.7 | 10.7 ± 0.7^ab^ | 10.6 ± 0.7^abc^ | <0.001 |
| Serum albumin (g/dL) | 4.02 ± 0.32 | 4.02 ± 0.32 | 4.01 ± 0.33^a^ | 3.99 ± 0.34^abc^ | <0.001 |
| Serum phosphorus (mg/dL) | 5.09 ± 1.23 | 5.02 ± 1.19^a^ | 4.96 ± 1.18^ab^ | 4.87 ± 1.23^abc^ | <0.001 |
| Serum calcium (mg/dL) | 8.86 ± 0.66 | 8.88 ± 0.67 | 8.90 ± 0.69^a^ | 8.88 ± 0.72 | 0.001 |
| Serum creatinine (mg/dL) | 9.74 ± 2.80 | 9.65 ± 2.65^a^ | 9.57 ± 2.58^a^ | 9.31 ± 2.53^abc^ | <0.001 |
| Use of RASB (%) | 8,050 (63.7) | 8,232 (65.1) | 8,342 (66.0) | 8,853 (70.0) | <0.001 |
| Use of aspirin (%) | 5,998 (47.4) | 6,219 (49.2) | 6,303 (49.9) | 6,164 (48.8) | 0.001 |
| Use of clopidogrel (%) | 3,357 (26.6) | 3,316 (26.2) | 3,300 (26.1) | 3,151 (24.9) | 0.019 |
| Use of statins (%) | 6,798 (53.8) | 6,541 (51.7) | 6,171 (48.8) | 5,681 (44.9) | <0.001 |
| Use of antihypertensive drug | 10,513 (83.2) | 10,612 (83.9) | 10,755 (85.1) | 11,069 (87.6) | <0.001 |
| MI or CHF (%) | 7,413 (58.6) | 7,335 (58.0) | 7,415 (58.7) | 7,403 (58.6) | 0.702 |
| Atrial fibrillation (%) | 1,554 (12.3) | 1,541 (12.2) | 1,578 (12.5) | 1,509 (11.9) | 0.609 |
| Dementia (%) | 1,003 (7.9) | 1,060 (8.4) | 1,043 (8.3) | 1,118 (8.8) | 0.070 |

Data are expressed as means ± standard deviations for continuous variables and numbers (percentages) for categorical variables. *P*-values were obtained using one-way analysis of variance, followed by Tukey’s post hoc test. Pearson’s χ^2^ test was performed for categorical variables. ^a^*P* < 0.05 versus aQ1, ^b^*P* < 0.05 versus aQ2, ^c^*P* < 0.05 versus aQ3.

**Abbreviations:** aQ1, first quartile of variability in weight-adjusted ultrafiltration volume; aQ2, second quartile of variability in weight-adjusted ultrafiltration volume; aQ3, third quartile of variability in weight-adjusted ultrafiltration volume; aQ4, fourth quartile of variability in weight-adjusted ultrafiltration volume; CCI, Charlson comorbidity index; CHF, congestive heart failure; MI, myocardial infarction; and RASB, renin–angiotensin system blocker.

**Table S8. Cox regression analyses according to quartiles of variability in weight-adjusted ultrafiltration volume.**

|  | **Univariable** | | **Multivariable** | |  |
| --- | --- | --- | --- | --- | --- |
|  | **HR (95% CI)** | ***P*-value** | **HR (95% CI)** | ***P*-value** | |
| **All-cause mortality** |  |  |  |  | |
| Ref: aQ1 |  |  |  |  | |
| aQ2 | 1.07 (1.03–1.12) | 0.003 | 1.02 (0.97–1.07) | 0.480 | |
| aQ3 | 1.14 (1.09–1.19) | <0.001 | 1.06 (1.01–1.11) | 0.025 | |
| aQ4 | 1.27 (1.21–1.33) | <0.001 | 1.16 (1.11–1.22) | <0.001 | |
| Ref: aQ2 |  |  |  |  | |
| aQ3 | 1.06 (1.01–1.11) | 0.011 | 1.04 (0.99–1.09) | 0.112 | |
| aQ4 | 1.18 (1.13–1.23) | <0.001 | 1.14 (1.09–1.20) | <0.001 | |
| Ref: aQ3 |  |  |  |  | |
| aQ4 | 1.12 (1.07–1.16) | <0.001 | 1.10 (1.05–1.15) | <0.001 | |
| **Cardiovascular events** |  |  |  |  | |
| Ref: aQ1 |  |  |  |  | |
| aQ2 | 1.08 (0.99–1.16) | 0.057 | 1.04 (0.96–1.13) | 0.327 | |
| aQ3 | 1.09 (1.01–1.18) | 0.025 | 1.09 (1.00–1.18) | 0.049 | |
| aQ4 | 1.15 (1.07–1.24) | <0.001 | 1.14 (1.05–1.24) | 0.003 | |
| Ref: aQ2 |  |  |  |  | |
| aQ3 | 1.01 (0.94–1.09) | 0.735 | 1.04 (0.96–1.13) | 0.310 | |
| aQ4 | 1.07 (0.99–1.15) | 0.067 | 1.09 (1.01–1.18) | 0.034 | |
| Ref: aQ3 |  |  |  |  | |
| aQ4 | 1.06 (0.98–1.14) | 0.135 | 1.05 (0.97–1.13) | 0.253 | |
| **Atrial fibrillation** |  |  |  |  | |
| Ref: aQ1 |  |  |  |  | |
| aQ2 | 0.99 (0.92–1.07) | 0.832 | 1.00 (0.92–1.08) | 0.930 | |
| aQ3 | 0.99 (0.92–1.07) | 0.871 | 1.01 (0.93–1.10) | 0.854 | |
| aQ4 | 1.02 (0.95–1.11) | 0.541 | 1.08 (0.99–1.18) | 0.079 | |
| Ref: aQ2 |  |  |  |  | |
| aQ3 | 1.00 (0.93–1.08) | 0.961 | 1.01 (0.93–1.10) | 0.783 | |
| aQ4 | 1.03 (0.96–1.12) | 0.407 | 1.08 (0.99–1.18) | 0.058 | |
| Ref: aQ3 |  |  |  |  | |
| aQ4 | 1.03 (0.96–1.11) | 0.434 | 1.07 (0.99–1.16) | 0.100 | |
| **Dementia** |  |  |  |  | |
| Ref: aQ1 |  |  |  |  | |
| aQ2 | 1.19 (1.08–1.32) | <0.001 | 1.15 (1.03–1.29) | 0.011 | |
| aQ3 | 1.24 (1.12–1.37) | <0.001 | 1.20 (1.07–1.34) | 0.002 | |
| aQ4 | 1.35 (1.22–1.49) | <0.001 | 1.31 (1.17–1.46) | <0.001 | |
| Ref: aQ2 |  |  |  |  | |
| aQ3 | 1.04 (0.94–1.14) | 0.484 | 1.04 (0.93–1.15) | 0.509 | |
| aQ4 | 1.13 (1.03–1.24) | 0.011 | 1.13 (1.02–1.26) | 0.020 | |
| Ref: aQ3 |  |  |  |  | |
| aQ4 | 1.09 (0.99–1.20) | 0.066 | 1.09 (0.99–1.21) | 0.087 | |

Multivariable analysis was adjusted for age; sex; body mass index; vascular access type; diabetes; hemodialysis vintage; Charlson Comorbidity Index score; ultrafiltration volume; Kt/V_urea_; levels of hemoglobin, serum albumin, serum creatinine, serum phosphorus, and serum calcium; use of renin–angiotensin system blockers, statins, clopidogrel, aspirin, or anti-hypertensive drugs; presence of myocardial infarction or congestive heart failure; and atrial fibrillation.

**Abbreviations**: aQ1, first quartile of variability in weight-adjusted ultrafiltration volume; aQ2, second quartile of variability in weight-adjusted ultrafiltration volume; aQ3, third quartile of variability in weight-adjusted ultrafiltration volume; aQ4, fourth quartile of variability in weight-adjusted ultrafiltration volume; CI, confidence interval; and HR, hazard ratio.

**Table S9. Baseline characteristics of Q1 and Q4 groups after propensity score matching**

|  | **Q1 (n = 10,925)** | **Q4 (n = 10,927)** | ***P*-value** |
| --- | --- | --- | --- |
| Age (years) | 61.7 ± 12.9 | 61.3 ± 12.9 | 0.061 |
| Sex (male, %) | 6,723 (61.5%) | 7,009 (64.1%) | <0.001 |
| Hemodialysis vintage (months) | 65 ± 69 | 65 ± 65 | 0.904 |
| Body mass index (kg/m^2^) | **22.8** ± 3.7 | 22.9 ± 3.6 | 0.761 |
| Diabetes (%) | 4,842 (44.3%) | 4,941 (45.2%) | 0.004 |
| CCI score | 8.8 ± 2.9 | 8.9 ± 2.8 | 0.353 |
| Arteriovenous fistula (%) | 9,433 (86.3%) | 9,443 (86.4%) | 0.886 |
| Kt/V_urea_ | 1.56 ± 0.27 | 1.55 ± 0.27 | 0.015 |
| Ultrafiltration volume (L/session) | 2.32 ± 1.02 | 2.34 ± 0.78 | 0.123 |
| Hemoglobin (g/dL) | 10.7 ± 0.7 | 10.7 ± 0.8 | 0.241 |
| Serum albumin (g/dL) | 4.01 ± 0.32 | 4.01 ± 0.34 | 0.592 |
| Serum phosphorus (mg/dL) | 5.00 ± 1.20 | 5.01 ± 1.27 | 0.602 |
| Serum calcium (mg/dL) | 8.9 ± 0.7 | 8.9 ± 0.7 | 0.790 |
| Serum creatinine (mg/dL) | 9.6 ± 2.7 | 9.7 ± 2.6 | 0.013 |
| Use of RASB (%) | 7,272 (66.6%) | 7,388 (67.6%) | 0.102 |
| Use of aspirin (%) | 5,253 (48.1%) | 5,361 (49.1%) | 0.151 |
| Use of clopidogrel (%) | 2,883 (26.4%) | 2,871 (26.3%) | 0.860 |
| Use of statins (%) | 5,469 (50.1%) | 5,372 (49.2%) | 0.189 |
| Use of antihypertensive drug | 9,323 (85.3%) | 9,424 (86.2%) | 0.057 |
| MI or CHF (%) | 6,394 (58.5%) | 6,361 (58.2%) | 0.649 |
| Atrial fibrillation (%) | 1,342 (12.3%) | 1,309 (12.0%) | 0.504 |
| Dementia (%) | 899 (8.2%) | 936 (8.6%) | 0.382 |

Data are expressed as means ± standard deviation for continuous variables and as numbers (percentages) for categorical variables. *P*-values were tested using student t-test. Pearson’s χ^2^ test was performed for categorical variables.

**Abbreviations:** Q1, first quartile of variability in ultrafiltration volume; Q2, second quartile; Q3, third quartile; Q4, fourth quartile; CCI, Charlson comorbidity index; CHF, congestive heart failure; MI, myocardial infarction; RASB, renin–angiotensin system blocker.

**Table S10. Cox regression analyses using propensity score matching**

|  | **Univariable** | | **Multivariable** | |  |
| --- | --- | --- | --- | --- | --- |
|  | **HR (95% CI)** | ***P*-value** | **HR (95% CI)** | ***P*-value** | |
| All-cause mortality | 1.12 (1.07–1.17) | <0.001 | 1.12 (1.07–1.18) | <0.001 | |
| Cardiovascular events | 1.15 (1.06–1.25) | <0.001 | 1.15 (1.06–1.26) | <0.001 | |
| Atrial fibrillation | 1.06 (0.98–1.15) | 0.170 | 1.11 (1.02–1.22) | 0.018 | |
| Dementia | 1.38 (1.24–1.54) | <0.001 | 1.39 (1.24–1.57) | <0.001 | |

Multivariable analysis was adjusted for age, sex, body mass index, vascular access type, diabetes, hemodialysis vintage, Charlson Comorbidity Index score, ultrafiltration volume, Kt/V_urea_, levels of hemoglobin, serum albumin, serum creatinine, serum phosphorus, and serum calcium, use of renin–angiotensin system blockers, statins, clopidogrel, aspirin, or anti-hypertensive drugs, presence of myocardial infarction or congestive heart failure, and atrial fibrillation. The values were hazard ratio of Q4 group compared to Q1 group.

**Abbreviations**: Q1, first quartile of variability in ultrafiltration volume; Q4, fourth quartile; CI, confidence interval; HR, hazard ratio.

**
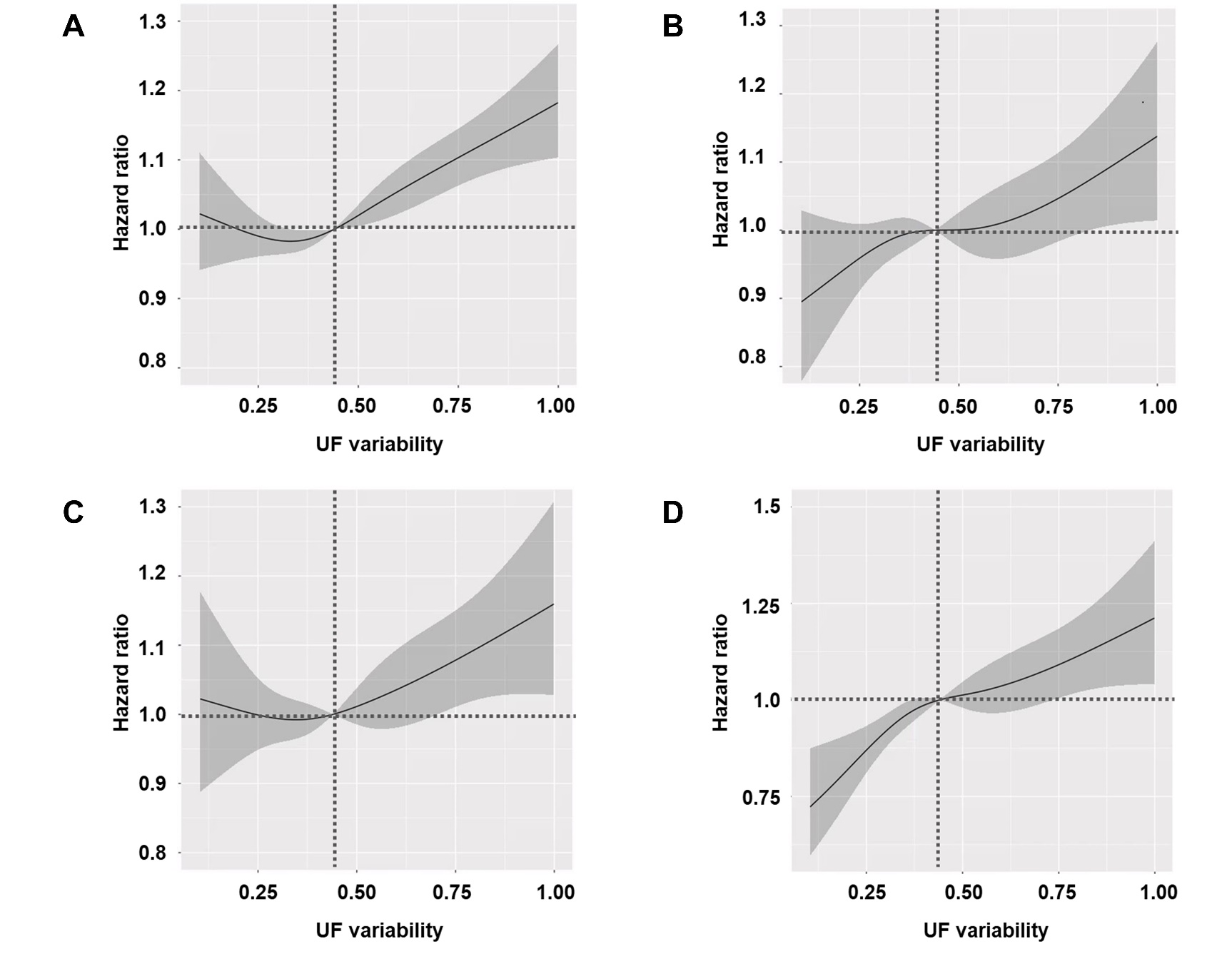
**

**Figure S1. Spline curves illustrating hazard ratios and 95% confidence intervals for clinical outcomes according to variability of UFV.** (A) All-cause mortality. (B) Cardiovascular events. (C) Atrial fibrillation. (D) Dementia. The reference point was established at 4.4 L/session of variability of UFV. Data were plotted using multivariable model, and adjustments were made according to the following factors: age; sex; body mass index; diabetes; type of vascular access; hemodialysis vintage; Charlson Comorbidity Index score; UFV; Kt/V_urea_; levels of hemoglobin, creatinine, phosphorus, and calcium; the use of renin–angiotensin system blockers, aspirin, clopidogrel, statins, or anti-hypertensive drugs; and the presence of myocardial infarction, congestive heart failure, or atrial fibrillation.

**Abbreviations:** UF, ultrafiltration volume


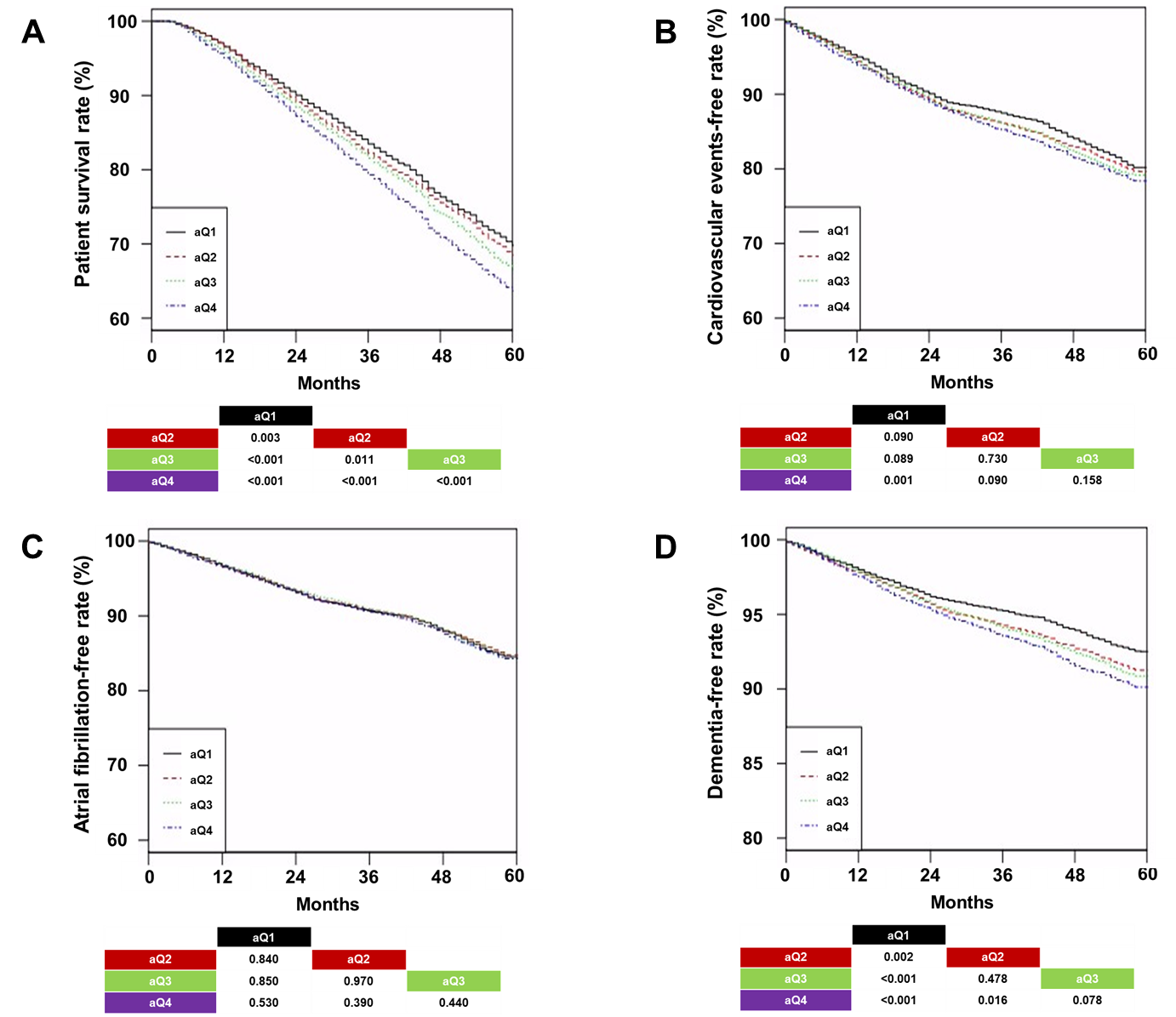


**Figure S2. Kaplan–Meier curves of patient survival, cardiovascular events, atrial fibrillation, and dementia according to quartiles of variability in weight-adjusted ultrafiltration volume.** (A) Patient survival. (B) Cardiovascular events. (C) Atrial fibrillation. (D) Dementia.

*P*-values for pairwise comparisons using log-rank tests have been added to the bottom of the graph.

**Abbreviations:** aQ1, first quartile of variability in weight-adjusted ultrafiltration volume; aQ2, second quartile of variability in weight-adjusted ultrafiltration volume; Q3, third quartile of variability in weight-adjusted ultrafiltration volume; and Q4, fourth quartile of variability in weight-adjusted ultrafiltration volume.

**
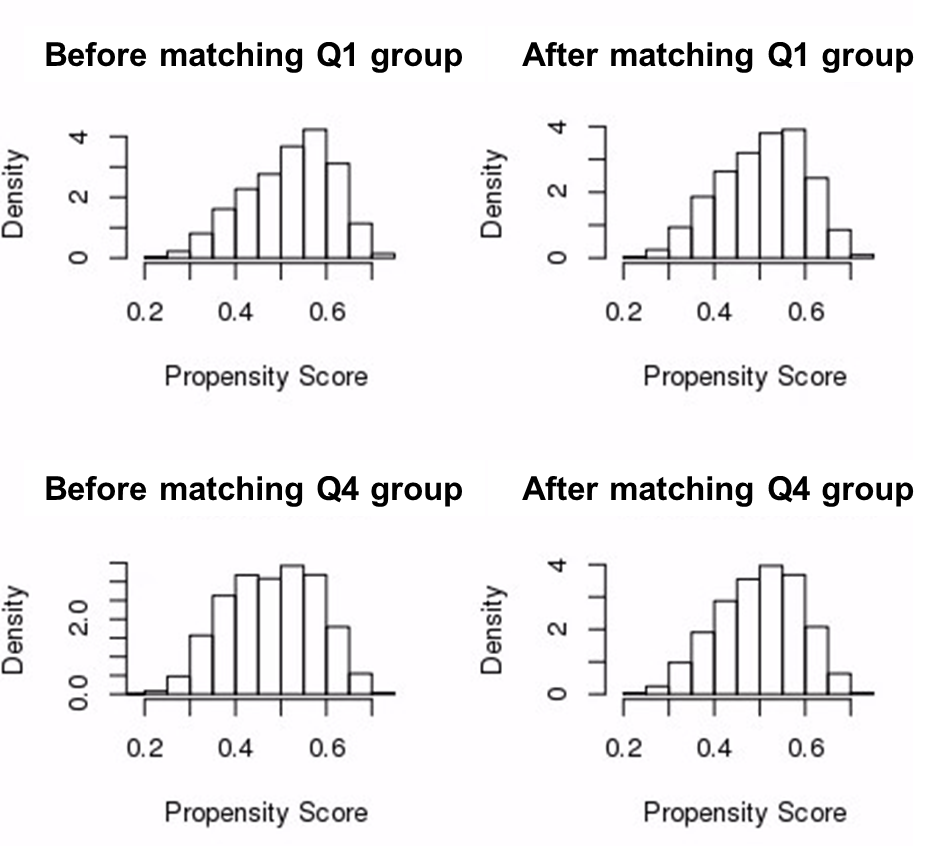
**

**Figure S3. Distribution of propensity scores before and after matching.** The distribution of propensity scores before matching differed between the Q1 and Q4 groups. However, there was no difference after matching.
